# *Burkholderia* Species Are the Most Common and Preferred Nodulating Symbionts of the Piptadenia Group (Tribe Mimoseae)

**DOI:** 10.1371/journal.pone.0063478

**Published:** 2013-05-15

**Authors:** Caroline Bournaud, Sergio Miana de Faria, José Miguel Ferreira dos Santos, Pierre Tisseyre, Michele Silva, Clémence Chaintreuil, Eduardo Gross, Euan K. James, Yves Prin, Lionel Moulin

**Affiliations:** 1 CIRAD, UMR LSTM, Montpellier, France; 2 EMBRAPA Agrobiologia, Seropédica, RJ, Brazil; 3 Depto de Ciências Agrárias e Ambientais, Universidade Estadual de Santa Cruz, Ilhéus, BA, Brazil; 4 IRD, UMR LSTM, Montpellier, France; 5 The James Hutton Institute, Dundee, United Kingdom; Graz University of Technology (TU Graz), Austria

## Abstract

*Burkholderia* legume symbionts (also called α-rhizobia) are ancient in origin and are the main nitrogen-fixing symbionts of species belonging to the large genus *Mimosa* in Brazil. We investigated the extent of the affinity between *Burkholderia* and species in the tribe Mimoseae by studying symbionts of the genera *Piptadenia* (*P.*), *Parapiptadenia* (*Pp.*), *Pseudopiptadenia* (*Ps.*), *Pityrocarpa* (*Py.*), *Anadenanthera* (*A.*) and *Microlobius* (*Mi.*), all of which are native to Brazil and are phylogenetically close to *Mimosa*, and which together with *Mimosa* comprise the “Piptadenia group”. We characterized 196 strains sampled from 18 species from 17 locations in Brazil using two neutral markers and two symbiotic genes in order to assess their species affiliations and the evolution of their symbiosis genes. We found that *Burkholderia* are common and highly diversified symbionts of species in the Piptadenia group, comprising nine *Burkholderia* species, of which three are new ones and one was never reported as symbiotic (*B. phenoliruptrix*). However, α-rhizobia were also detected and were occasionally dominant on a few species. A strong sampling site effect on the rhizobial nature of symbionts was detected, with the symbiont pattern of the same legume species changing drastically from location to location, even switching from β to α-rhizobia. Coinoculation assays showed a strong affinity of all the Piptadenia group species towards *Burkholderia* genotypes, with the exception of *Mi. foetidus*. Phylogenetic analyses of neutral and symbiotic markers showed that symbiosis genes in *Burkholderia* from the Piptadenia group have evolved mainly through vertical transfer, but also by horizontal transfer in two species.

## Introduction

Legumes have developed a symbiosis with a polyphyletic group of bacteria commonly called rhizobia. This symbiosis leads to the formation of a specialized organ, the root nodule, within which rhizobia differentiates into bacteroids. Bacteroids fix atmospheric nitrogen, and feed the plant with combined nitrogen in exchange for carbon compounds derived from photosynthesis by the legume host. Over the last several decades, numerous diversity studies have focused on rhizobia, but their diversity and the number of investigated legumes hosts remain far from being complete due to the large number of legume species (>18000) [1].

Most rhizobia belong to a large diversity of alphaproteobacterial genera: Azorhizobium, Allorhizobium, Bradyrhizobium, Mesorhizobium, Rhizobium, Sinorhizobium (Ensifer), Devosia, Methylobacterium, Ochrobactrum, Phyllobacterium, and more recently Aminobacter [2] and Microvirga [3], whereas Burkholderia and Cupriavidus are members of the betaproteobacteria [4], [5], [6], [7]. The terms α and β-rhizobia have thus been raised to distinguish each class of symbionts [7], [8]. Burkholderia is a highly diversified genus, including more than 70 species that have colonised a wide diversity of niches, ranging from soil and water to plants and animals [9], [10].

Diversity studies of rhizobia and legume host range have shown that the vast majority of nodulating legume species interact with α-rhizobia [7]. To date, β-rhizobia are much more restricted in terms of host range, and most species described so far interact with *Mimosa* species in their major area of diversification in central Brazil and other parts of the tropical World (for a review see [7]). *Mimosa* species symbionts include mainly *Burkholderia* species, such as *B. tuberum* [11], [12], *B. mimosarum* [13], [14], [15], [16], *B. phymatum*, [12], [16], [17], *B. nodosa* [18], *B. sabiae* [19], *B. symbiotica* [20], *B. diazotrophica* [21], and two species of *Cupriavidus*: *C. taiwanensis* [5] and *C. necator* [22]. Interestingly, further studies have shown that nodulation by *Burkholderia* could be extended to other legumes, such as some native/endemic African and Australian species in the subfamily Papilionoideae [23], [24]. For example, *B. tuberum* STM678 nodulates many *Cyclopia* species [23], and it harbors distinct nodulation genes compared to *Mimosa*-nodulating burkholderia, suggesting South African and South American burkholderias acquired their nodulation genes in distinct transfer events [8], [14], [12], [7].

The relationship between β-rhizobia and *Mimosa* spp. was investigated in more depth in two studies reported by [11] and [25]. These authors analyzed the diversity of symbionts from nodules of c. 70 diverse *Mimosa* species growing in the two major biomes of Brazil (Cerrado and Caatinga) in which the genus *Mimosa* has evolved and diversified into more than 200 native and endemic species [26]. They concluded on the generic character of nodulation in the genus *Mimosa* that the preferred symbionts of this genus in Brazil are *Burkholderia*. Identical tree topologies between neutral and symbiotic markers were also observed on Brazilian and French Guianan *Mimosa-*nodulating burkholderia [11], [12], indicating a monophyletic origin and single acquisition of symbiotic genes by a *Burkholderia* ancestor, followed by vertical transfer of nodulation genes during species diversification [11]. On the other hand, based on phylogenies of nodulation genes, *Cupriavidus taiwanensis* was found to be a more recent symbiont of *Mimosa* spp., which acquired nodulation genes from a *Burkholderia* ancestor [8], [12], [27]. Although most species of *Mimosa* are mainly nodulated by *Burkholderia* spp., and a few by *Cupriavidus* spp., some of them can also form effective symbioses with α-rhizobia [8], [11], [28], [29]. Later studies have underlined the existence of genetic and environmental factors that could affect the preference of legumes species for nodulation with either α- or β-rhizobia; these include soil pH or the presence of combined nitrogen [11], [12], [16], [24], [25].

The *Mimosa* genus is closely related to a number of genera including *Piptadenia (P.)*, *Parapiptadenia (Pp.)*, *Pityrocarpa (Py.)*, *Anadenanthera (A.)*, *Stryphnodendron (St.)*, and *Microlobius (Mi.)* (syn. *Goldmania*) within the tribe Mimoseae. Jobson & Luckow [30] have investigated the phylogeny of these different genera and subdivided the *Piptadenia* genus into three clades: the Piptadenia genus *sensu stricto* (or Eupiptadenia clade) that is a sister clade to *Mimosa*, and includes, for example *P. flava* (type species), *P. floribunda*, *P. gonoacantha*, *P. paniculata*; the Pityrocarpa clade (*Py. leucoxylon*, *Py. monoliformis*, *Py. obliqua*) that is closer to the genera *Stryphnodendron* and *Parapiptadenia* than to the Eupiptadenia clade; and finally *P. viridiflora* that is outgrouped from the previous genera and represent a particular case deserving a new generic name [30]. Interestingly, all these genera contain woody species native to South America, particularly to Brazil [31], [30]. Some of them are currently exploited by locals owing to their economical values, such as *A. peregrina* [32], [33], *Pp. rigida* [34], [35] and *P. gonoacantha* [36]. Although their ability to establish associations with rhizobia is documented [37], [38], [39], [40], [41], [42], [43], information about the rhizobial diversity and symbiotic efficiency on these plant species is scarce. A recent study of symbionts of *Parapiptadenia rigida* in Uruguay demonstrated that this species is nodulated by rhizobial strains belonging to the genera *Burkholderia*, *Cupriavidus* and *Rhizobium*, among which the *Burkholderia* genotypes were the predominant group [44]. Symbiosis with β-rhizobia in the tribe Mimoseae thus appears to extend outside *Mimosa* and be more common than previously expected.

In this study our objectives were (i) to investigate the extent of *Burkholderia* affinity within the tribe Mimoseae by focusing on native species in the *Piptadenia* genus and in related genera in the Piptadenia Group described by Jobson & Luckow [30]; (ii) to characterize the diversity of rhizobia and their symbiotic genes in this group of legumes; (iii) to examine further symbiotic specificity within the Piptadenia Group.

To achieve these objectives, we isolated rhizobia from diverse species in the Piptadenia Group in their native areas in Brazil, characterized their taxonomic and symbiotic diversity, and assessed their host specificity via coinoculation assays. We found a large diversity of *Burkholderia* species, but also α-rhizobia, and with a few exceptions an affinity of most plant species towards *Burkholderia* rather than to α-rhizobia. We discuss the evolutionary patterns of both taxonomic and symbiotic markers and the putative co-adaptation between *Burkholderia* and Piptadenia Group species.

## Materials and Methods

### Bacterial Isolates Sampling and Maintenance

**Figure 1:**
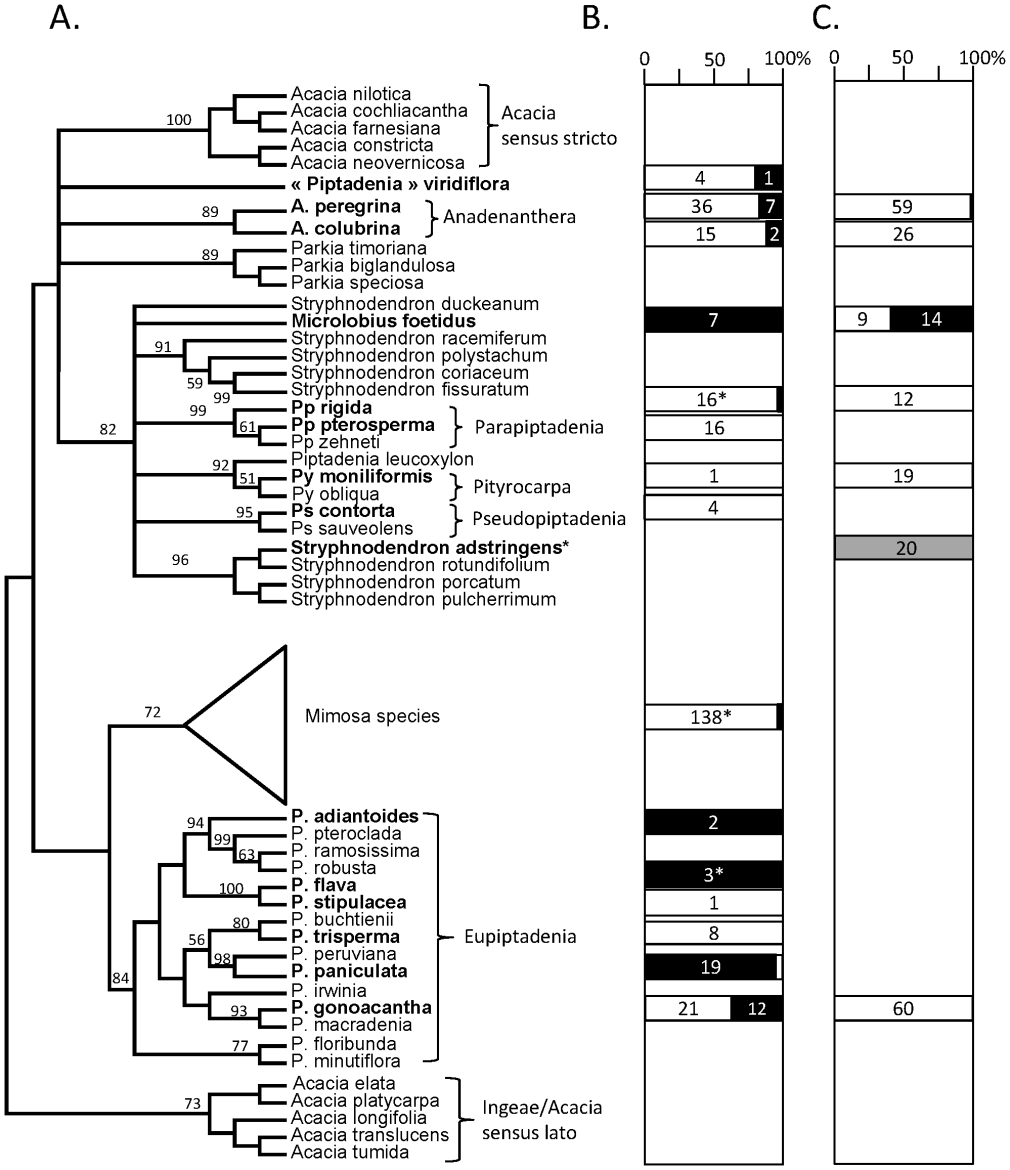
Comparison between Piptadenia’s group plant phylogeny and the occurrence of alpha and beta-rhizobia as nodule symbionts in the field or during coinoculation experiments. The plant phylogeny (A) is based on a trnL-F/trnK-matK combined dataset, and was built by parsimony with TNT1.1 (default parameters, on www.phylogeny.fr) using the Jobson & Luckow [30] dataset (downloaded from Treebase, study number S1763, and amended with the *P. trisperma* from this study). The % of α and β-rhizobia per legume host (in bold) from field sampling (B) or from the coinoculation experiment (C) are represented as white (*Burkholderia*) and black (α-rhizobia) squares, with the number of strains sampled within each square. *: statistics of symbionts from [44] and [11]. The grey colored square for *Stryphnodendron* indicates that % of α-rhizobia originates from a trapping experiment on soil (see Material & Methods section).

Collection of material was authorized by IBAMA N°. 058/2006. The bacterial collection was built through several sampling campaigns performed between 1984 and 2010, from 9 states in Brazil (Rio de Janeiro, Sao Paulo, Minas Gerais, Bahia, Mato Grosso do Sul, Paranà, Pernambuco, Espirito Santo and Pará). Sample locations were concentrated on the main centers of diversification of the Piptadenia Group (Mata Atlantica) and are presented in Figure S1. Details on sampling (gps coordinates, year, season, soil type and pH) are presented in Table S1. STM strains were sampled from trees from various places of Rio de Janeiro State in April 2010; SFM, BR and CVRD strains were sampled from plants growing in plant nursery beds in various states of Brazil (Table S2). Nodules sampled from the field as described in [37] were dried on silicagel until symbiont isolation. Nodules were then rehydrated for 30 min in sterile distilled water, surface sterilized by immersion 30 seconds in 70% ethanol followed by 1 to 3 min in 3% hydrogen peroxide, and washed three times in sterile distilled water. Nodules were then individually crushed and streaked on yeast mannitol agar (YMA, [45]) plates containing bromophenol blue. All YMA plates were incubated at 28°C. Single colonies were picked and checked for purity by repeated streaking and microscope examination. All pure isolates were stored at −80°C in YM broth plus 20% (w/v) glycerol. For *Stryphnodendron* species, no nodules could be found on two locations, so a plant trapping approach was developed by growing seedlings on a top soil harvested under each tree, and nodules were harvested and processed as described above. As these rhizobia were trapped, they were not treated as natural symbionts, and were only included on Figure 1C to assess rhizobial patterns among the Piptadenia group. Strains from previous studies on *P. flava* [42], *Pp. rigida* [44], [46], as well as *P. gonoacantha* and *Pp. pterosperma* [46] were also included in this study. A total of 196 strains were included in the study, of which 63 “representative” strains are listed in Table 1 and were chosen as one strain per host plant per geographical origin per unique 16 S rDNA ribotype (as described in molecular method section).

**Table 1:** Listing of strains used in this study.

Original Host/Strain	Ribotype^%^		nodCclade				Bacterialspecies&			GeographicalOrigin and source							Nb£			Nod#				
***Piptadenia gonoacantha***																								
STM7321	R1		C12				*Rhizobium* sp. 1			2 (RJ), this study							4			Pg, Sir				
STM7300	R1		C12				*Rhizobium* sp. 1			2 (RJ), this study							15			Pg, Sir				
SMF1181_6	R1		NT				*R. tropici*			17 (SP), this study							2			NT				
STM7315	B1		C3				*B. sabiae*			2 (RJ), this study							2			Pg, Mp, Pm, Ap, Ac, Ppr				
STM7319	B1		C3				*B. sabiae*			2 (RJ), this study							1			Pg, Mp, Sir				
SMF1181_1	B2		NT				*B. nodosa*			17 (SP), this study							1			NT				
P. gonoacantha_1	B3		NT				*B. nodosa*			16 (SP), this study							2			Pg				
P. gonoacantha_3	B4		NT				*Burkholderia* sp. 3			16 (SP), this study							2			Pg				
P. gonoacantha_8	B5		C2				*Burkholderia* sp. 3			16 (SP), this study							1			Pg				
STM7296	B6		C2				*Burkholderia* sp. 3			4.2 (RJ), this study							1			Pg, Mp				
STM7317	B7		C4				*B. phenoliruptrix*			2 (RJ), this study							1			Pg, Mp, Mf, Ap, Ac, Ppr, Pm				
BR4812	B8		NA				*B. diazotrophica*			[46] £							1			Pg				
***Piptadenia trisperma***																								
STM7351	B1		C1				*B. sabiae*			5 (RJ), this study							2			Mp				
STM7353	B9		C5				*B. nodosa*			5 (RJ), this study							4			Mp, Sir, Pg, Mf, Pm, Ap, Ac, Ppr				
STM7348	B2		C5				*B. nodosa*			5 (RJ), this study							2			Mp, Sir				
***Piptadenia paniculata***																								
STM7339	R2		NA				*R. tropici*			4.1 (RJ), this study							1			Nod- on Mp&Sir				
STM7342	R3		NA				*Rhizobium* sp. 3			4.1 (RJ), this study							1			Nod- on Mp&Sir				
STM7330	R1		C12				*Rhizobium* sp. 1			4.1 (RJ), this study							1			Pg, Sir				
STM7333	R4		C9				*Bradyrhizobium* sp. 1			4.1 (RJ), this study							1			Sir				
STM7334	R5		C9				*Bradyrhizobium* sp. 1			4.1 (RJ), this study							1			Sir				
STM7331	R6		C9				*Bradyrhizobium* sp. 3			4.1 (RJ), this study							5			Sir				
STM7332	R7		C11				*Bradyrhizobium* sp. 1			4.1 (RJ), this study							6			Sir				
STM7329	R8		C9				*Bradyrhizobium* sp. 3			4.1 (RJ), this study							3			Sir				
STM7324	B7		C4				*B. phenoliruptrix*			4.1 (RJ), this study							1			Mp, Sir, Pg				
***Piptadenia adiantoides***																								
SMF1758_4	R9		NT				*Rhizobium* sp. 6			10 (MG), this study							2			Pa				
SMF1758_8	R1		NT				*R. tropici*			10 (MG), this study							1			Pa				
***Piptadenia monoliformis***																								
SMF774_1	B10		C3				*B. phenoliruptrix*			3 (RJ), this study							1			Mp				
***Piptadenia viridiflora***																								
SMF1356_6	R1		C12				*Rhizobium* sp. 1			12 (BA), this study							4			NT				
SMF1356_7	B1		C3				*B. sabiae*			12 (BA), this study							1			NT				
JPY570 (CAE9)	B11		C3				*Burkholderia* sp. 1			Bahia, Gross et al.§							1			Pv, Pg, Mp				
JPY565 (CAE1)	B11		C3				*Burkholderia* sp. 1			Bahia, Gross et al.§							1			Pv, Pg, Mp				
***Piptadenia stipulacea***																								
JPY584 (D84)	B12		C3				*Burkholderia* sp. 4			Bahia, Gross et al.§							1			Ps, Pg, Mp				
***Piptadenia flava***																								
UPRM8060	R10		C8				*R. gallicum*			[42]							1			Pf				
UPRM8061T1	R10		C8				*R. gallicum*			[42]							1			Pf				
***Anadenanthera peregrina***																								
STM7420	R1		NT				*Rhizobium* sp. 1			2 (RJ), this study							1			Nod- Mp & Sir				
STM7426	R1		NT				*R. tropici*			2 (RJ), this study							1			Nod- Mp &Sir				
SMF466_6	R1		C12				*R. leucaenae*			6 (MGS), this study							5			NT				
IIIA_10R	B13		C3				*B. sabiae*			16 (SP), this study							1			NT				
STM7419	B1		C3				*B. sabiae*			2 (RJ), this study							1			Mp				
STM7384	B1		C3				*B. sabiae*			2 (RJ), this study							6			Mp				
SMF362_15	B2		C5				*B. nodosa*			8 (PA), this study							1			NT				
STM7399	B8		C3				*B. diazotrophica*			2 (RJ), this study							5			Ap, Mp				
SMF362_13	B14		NT				*B.* c*aribensis*			8 (PA), this study							3			NT				
IIIA_4A	B10		C3				*B. phenoliruptrix*			16 (SP), this study							1			NT				
STM7437	B7		C4				*B. phenoliruptrix*			2 (RJ), this study							15			Mp				
STM7415	B7		C4				*B. phenoliruptrix*			2 (RJ), this study							3			Mp				
***Anadenanthera colubrina***																								
AngicoI_417	R1		C12				*Rhizobium* sp. 2			7 (PE), this study							2			NT				
STM7444	B15		C3				*B. diazotrophica*			2 (RJ), this study							1			Nod- on Mp,Sir				
STM7439	B16		C3				*B. diazotrophica*			2 (RJ), this study							2			Mp				
STM7445	B17		C3				*B. diazotrophica*			2 (RJ), this study							3			Mp				
STM7443	B18		NT				*B. diazotrophica*			2 (RJ), this study							3			NT				
STM7452	B19		C3				*B. diazotrophica*			2 (RJ), this study							1			Mp				
STM7454	B7		C4				*B. phenoliruptrix*			2 (RJ), this study							5			Mp				
***Parapiptadenia pterosperma***																								
STM7365	B1	NT					*B. sabiae*			5 (RJ), this study							1			Pg				
STM7373	B1	C1					*B. sabiae*			5 (RJ), this study							1			Mp, Pg, Mf, Ap, Ac, Pm				
CVRDII_2	B20	C3					*B. phymatum*			15 (ES), this study							1			Pppt				
STM7363	B9	C5					*B. nodosa*			5 (RJ), this study							5			Mp, Sir, Pg				
STM7358	B2	C5					*B. nodosa*			5 (RJ), this study							2			Mp, Sir				
BR9001	B2	C5					*B. nodosa*			15 (ES), [46] £							1			Pppt				
BR9002	B9	C5					*B. nodosa*			15 (ES), [46] £							2			Pppt				
SMF142_3	B2	NT					*B. nodosa*			11 (MGS), this study							4			NT				
BR9003		C5					*B. nodosa*			[46] £							1			Pppt				
***Parapiptadenia rigida***																								
P. rigida_2	B1	C1					*B. sabiae*			16 (SP), this study							3			NT				
UYPR3.611		C1					*B. sabiae*			Uruguay, [44]							1			Ppr				
UYPR1.313		C1					*B. caribensis*			Uruguay, [44]							1			Ppr				
UYPR7.63		C13					*R. mesoamericanum*			Uruguay, [44]										Ppr				
BR9004		C5					*B. nodosa*			[46] £							1			Ppr				
***Parapiptadenia blanchetti***																								
EG100	B21	C3					*B. diazotrophica*			14 (BA), Gross et al.§							1			Mp				
***Microlobius foetidus***																								
STM7379	R1	C12					*R. tropici*			1 (RJ), this study							2			Mf, Ap				
STM7378	R6	NA					*Bradyrhizobium* sp.4			1 (RJ), this study							4			Mf, Sir				
STM7375	R8	C10					*Bradyrhizobium* sp.2			1 (RJ), this study							1			Sir				
***Pseudopiptadenia contorta***																								
CVRDIII_5	B3	C5			*B. nodosa*					15 (ES), this study							2			Psc				
CVRDIII_7	B2	C5			*B. nodosa*					15 (ES), this study							2			Psc				
***Pseudopiptadenia psilostachya***																								
SMF613_4	R1	NT			*R. tropici*					9 (Parà), this study							3			NT				
***Pseudopiptadenia bahiana***																								
EG118	B1	C1			*B. sabiae*					13 (BA), Gross et al.§							1			Mp				
***Stryphnodendron*** ** sp. (trapping)**																								
STM9027	R11	NT			*Bradyrhizobium* sp.					18 (RJ), this study							1			Str				
STM9026	R12	NT			*Bradyrhizobium* sp.					18 (RJ), this study							4			Str				
STM9018	R13	NT			*Bradyrhizobium* sp.					18 (RJ), this study							15			Str				

Symbols : ^%^ ribotype number as defined in Mat&Methods.

**Figure 2:**
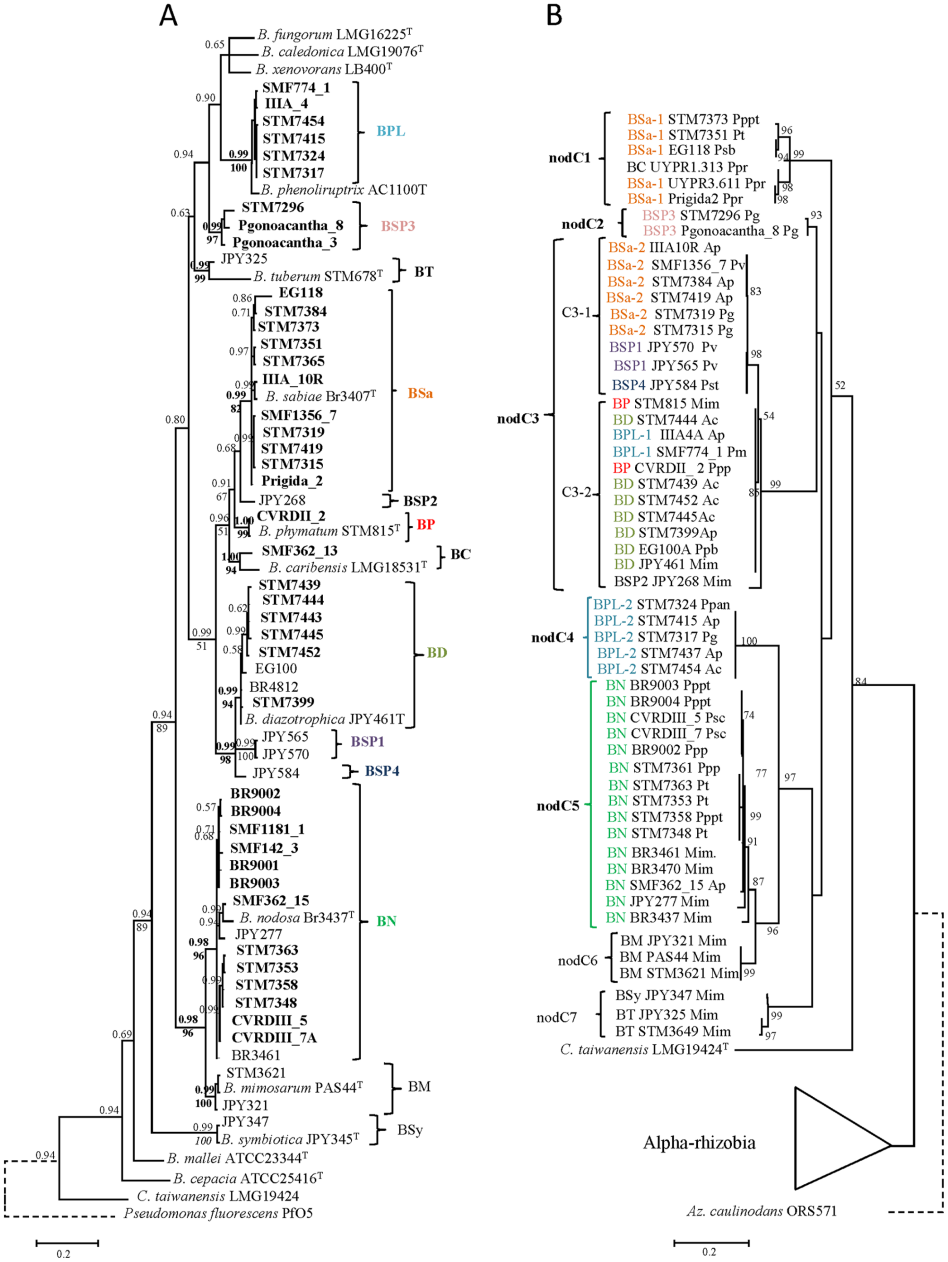
Comparison of phylogenies between neutral and symbiotic markers in beta-rhizobia. The neutral marker phylogeny (A) is based on a partition of 800 bp of 16 S rDNA and 600 bp of *recA* genes, and was built by a Bayesian analysis with priors estimated by ML (see Experimental procedure section). Numbers at ach nodes indicates posterior probabilities (upper number) and bootstraps values % (lower number) from a ML analyses built in parallel (with a GTR+I+G model, 1000 bootstrap replicates). Bootstraps are only indicated when >50%. Node values in bold indicates supported nodes (both by posterior probabilities and bootstrap) retained for clades delineation. The *nodC* phylogeny is based on 437 bp alignments, and was built by ML using a GTR+I+G model, with 1000 bootstraps replicates (% indicated at tree nodes if >50%). Abbreviations used: B.: *Burkholderia*, C.: *Cupriavidus*, BPL: *Burkholderia phenoliruptrix*, BT: *B. tuberum*, BSa: *B. sabiae*, BD: *B. diazotrophica*, BSy: *B. symbiotica*, BM: *B. mimosarum*, BN: *B. nodosa*, BSP1,2,3,4: *Burkholderia* sp. 1 to 4, ^T^ indicates species type strains. Accession numbers are listed in Table S3.

& Species affiliation based on the 16S-recA phylogeny from Figure 2A.

£ number of isolates from the same host, location and 16 S haplotype per representative strain listed in the first column.

# positive nodulation tests obtained from the representative strain.

§ Gross et al., unpublished g001data. £ Strains isolated from a different study but that were identified by molecular typing in this study. Abbreviations: B: *Burkholderia*, R: *Rhizobium*, Br: *Bradpone.0063478.g004.tifyrhizobium*, RJ: Rio de Janeiro, SP: Sao Paulo, Pg: *Piptadenia gonoacantha*, Pf: *Piptadenia flava*, Pm: *Pityrocarpa monoliformis*, Sir: Siratro (*Macroptilium atropurpureum*), Mp: *Mimosa pudica*, Psb: *Pseudopiptadenia bahiana*, Psc: *Ps. contorta*, Str: *Stryphnodendron* sp., Mf: *Microlobius foetidus*, Ppr: *Parapiptadenia rigida*, Pppt: *Pp. pterosperma*, Ap: *Anadenanthera peregrina*, Ac: *A. colubrina*, unp: unpublished, NT: not tested, NA: not amplified. Geographical origins numbering corresponds to: 1, Seropedica (Rio de Janeiro-RJ) (22° 23′ 95 “S/41° 49′23 O); 2, Seropedica nursery (RJ) (22° 44′38″ S/43° 42′28″ O); 3, Centro Nacional e Pesquisa Agropecuaria, Seropedica (RJ) Seropedica (RJ); 4.1, Cabo Frio (RJ) (22° 45′ 34″ S/41° 57′ 89″ O); 4.2, Cabo Frio (Rio de Janeiro) (22° 26′ 42″ S/41° 51′ 41″ O); 5, Cabo Frio – Bùzios (RJ) (22° 47′ 98″ S/41° 58′ 03″ O); 6, Corumba (Mato Grosso do Sul-MGS); 7, Embrapa semiàrido, Recife (Pernambuco-PE); 8, Telemaco Borba, Fazenda Monte alegre (Paranà-PA); 9, Estrada terra km 1 de guarita, Porto trombetas (Parà); 10, Mariana (Minas Gerais-MG); 11, Parque estudual do Rio doce, Marlièria (Minas Gerais); 12, Xique-Xique (Bahia-BA); 13, Jussari (BA) (15°16′46′′ S, 39°49′93′′ W); 14, Itaju do Colonia (BA) (15°15′99′′ S, 39°62′12′′ W); 15, Reserva Florestal da Cia, Linhares (Espirito Santo-ES); 16, Instituto Florestal de Sâo Paulo (Sâo Paulo-SP); 17, Paraibuna (SP); 18, Paracambi (RJ), 22°34′49.92′′S/43°41′13.90′′O.

### Plant Nodulation and Specificity Tests

Seeds of *P. gonoacantha*, *Py. monoliformis*, *Mi. foetidus* and *A. colubrina* were obtained from Instituto Brasileiro de Florestas (Londrina, Paraná Brasil) or Embrapa Agrobiologia (Seropédica; Brazil). *M. pudica* and siratro seeds were obtained from B&T World Seeds (Paguignan, France) and from University Cheikh Anta Diop (Dakar, Senegal), respectively. *P. gonoacantha* seeds were scarified and surface sterilized using 96% H_2_SO_4_ and 3% calcium hypochlorite (2 min and 3 min, respectively), while *A. colubrina, A. peregrina*, and *Pp. rigida* seeds were immersed for 3 min in 3% calcium hypochlorite; and *Mi. foetidus* seeds were sterilized with 96% H_2_SO_4_ during 30 min. *Py. monoliformis* seeds were germinated by immersion in concentrated H_2_SO_4_ for 5 min, then washed with sterile dH_2_O and afterwards were soaked in 3% calcium hypochloride for 3 min. All species seeds were then washed four times with sterile dH_2_O, before being germinated on 0.8% water agar plates at 28°C in the dark. *M. pudica* and siratro seeds were sterilized and germinated as described in [12].

For nodulation tests, all species were grown in Gibson tubes containing Jensen medium [45] filled with dH_2_O, except *Piptadenia* species for which tubes were filled with sterile attapulgite (OIL DRI US Special, IIIR, Damolin) and supplemented with sterile dH_2_O. All plants were then grown in a chamber at 26°C (relative humidity 40%) with a 16 h light/8 h night cycle. Inoculation was performed by adding 1 ml of exponential bacterial culture grown in broth YM medium. Cross-contamination was investigated by using uninoculated negative controls randomly placed between treatments. Plants were observed for nodulation over a period of 30–60 dai, depending on plant species. Nitrogen fixation was estimated by visual observation of plant vigour and foliage color.

For plant-rhizobium coinoculation experiments, plants were grown in Gibson tubes filled with attapulgite, moistened with sterile water and inoculated with 1 ml of an equal mixture of 12 representative strains calibrated at 10 e^6^ cells using KOVA slides microscope counting. Plants were incubated in the same conditions than as described above, watered with dH_2_O and harvested 4 weeks post inoculation. Eight nodules were harvested per nodulated plant (12 replicates), surface sterilized 3 min with 3% calcium hypochlorite, placed in a microplate, crushed using a replicator, grown on YMA microplates, and genotyped on *recA* gene marker as described in the molecular methods section.

### Molecular Methods

Extraction of genomic DNA of bacterial strains was performed using the proteinase K lysis procedure described previously [47]. PCR amplifications were performed with GO-Taq Polymerase (Promega) following the manufacturer instructions. All PCR templates were generated with specific primer sets listed in Table S2. 16 S rDNA amplification was carried out as previously described [12]. A 800-bp *recA* fragment was amplified by PCR and sequenced using the primers recABurk1F and recABurk1R for β-rhizobia, as described in [11], while two specific couples of primers were used for *Bradyrhizobium* (TSrecAf and TSrecAr) and *Rhizobium* (recAf and recAr) using PCR conditions described in [48] and [49], respectively. A 440-bp fragment of *nifH* was amplified and sequenced on *Burkholderia* strains as described in [8], and on *Rhizobium* and *Bradyrhizobium* strains as described in [50]. A 600-bp fragment of *nodC* was amplified and sequenced for *Burkholderia* strains using primers described in [11] except for BSP1, BD, BPL and BSa strains for which a specific primer set (nodCPipF and nodCPipR) was designed (500 bp fragment). For α-rhizobia, 600 bp of *nodC* for *Bradyrhizobium* and 800 bp for *Rhizobium* strains were amplified with primer sets nodCfor540-nodCrev1560 and nodCF-nodCI respectively [51], [50]. DNA sequencing was subcontracted to Genoscreen Inc., using ABI3730 sequencers, and the same primers as used for PCR, except for partial 16 S rRNA sequencing for which an internal primer was used (16 S–1080 r).

For the identification of the rhizobial nodule occupant from the plant coinoculation experiment, DNA from surface-sterilized crushed nodule were heat-shocked in 20 µl using a PCR machine (1 cycle of 2 min at 96°C, followed by 7 cycles of 10 seconds at 96°C then 4°C, with a final cycle of 2 min at 4°C), and 1 µl was used to amplify a *recA* gene fragment as described above. PCR templates were sequenced as previously described.

Plant phylogenetic markers trnL-F and trnk-matK of *Stryphnodendron* sp. and *P. trisperma* were PCR-amplified and sequenced as described by [30], from leaves collected from the field.

### Phylogenetic and Sequence Analyses

Nucleotide sequences from 16 S rDNA, *recA*, *nodC* and *nifH* genes were corrected with CHROMAS PRO v1.33 (Technelysium Pty Ltd), aligned using Muscle3.6 [52], and alignments were manually curated with GENEDOC [53]. Screening and classification of each 16 S rDNA haplotype (unique sequence in our dataset) was performed using MOTHUR using the unique.seqs command [54]. Phylogenetic trees were constructed by neighbor joining and likelihood methods using MEGA5 [55] and PAUP4 [56], or by Bayesian analyses using Mr Bayes [57] using priors from a GTR+I+G model (with parameters previously estimated by ML under PAUP4). Parsimony analyses on trnL*-*F*+*trnK*-*matK plant markers were performed on TNT1.1 (default parameters, www.phylogeny.fr) using the Jobson & Luckow [30] dataset (downloaded from Treebase, www.treebase.org, study number S1763), amended with new sequence from this study (*P. trisperma*). Bootstrapping analyses were conducted on MEGA5.

### Nucleotide Sequence Accession Numbers

The sequences have been deposited in EMBL database under accession numbers HE983632 to HE983823 and HF536727 to HF536767, and are listed by gene for each strain in Table S3. Sequence alignments are available upon request.

## Results

### Building a Collection of Rhizobia from Piptadenia Group Species

We sampled at least one representative species of the different clades and genera in the Piptadenia Group (see species in bold in Figure 1A): *Piptadenia sensu stricto* (the “Eupiptadenia” clade e.g. *P. gonoacantha*, *P. paniculata*, *P. adiantoides*), the Pityrocarpa clade (*Py. moniliformis*), the *P. viridiflora* clade, and the *Anadenanthera*, *Parapiptadenia*, *Stryphnodendron* and *Pseudopiptadenia* clades. The species assignment of each plant host was confirmed by the Botanical garden of Rio de Janeiro (R. Ribeiro). For five species (*Stryphnodendron adstringens*, *P. trisperma*, *P. paniculata*, *P. gonoacantha* and *A. peregrina*), the taxonomic position was confirmed by sequencing the trnL-F marker, and comparing it to the Jobson & Luckow dataset [30]. Among all sampled legume species, we confirmed that 17 of them are nodulated, and one, *Piptadenia trisperma* is a new report for nodulation (according to nodulation data in GRIN database, http://www.ars-grin.gov/~sbmljw/cgi-bin/nodulation.pl and [58]). The rhizobial collection was composed of 196 isolates and details are presented in Table 1, where they are classified according to original host legume, representative strain and site of isolation. Representative strains for each location and legume host were chosen according to their 16 S rDNA sequence haplotype (named ribotype in Table 1): one strain per legume host and per sampling location with a unique 16 S rDNA sequence based on a 800 bp alignment was kept for further analyses, and designated as representative strains (for a total of 63 for strains from this work, and 79 when including reference strains from other studies).

Nodulation ability of the representative strains was confirmed either on their original host or on *M. pudica* and siratro when seeds of the original host could not be obtained (see Table 1, Nodulation column), except for 15 strains which were not tested. No nodulation was observed on *M. pudica* or siratro by five strains (STM7339, STM7342, STM7420, STM7426, STM7444), but these strains were kept as the original host could not be tested. Details about nodule size, shape and functionality for each *Burkholderia* species are given in Table S4 and Figure S2, together with pictures of nodules on *M. pudica* and *P. gonoacantha*. All nodulating strains appeared to fix nitrogen with *M. pudica* (based on red color within nodules usually linked to presence of leghaemoglobin, and plant development, see Table S4), except some *B. sabiae* (carrying nodC1 variant) and *B. nodosa* (nodC5 variant) strains that produced ineffective nodules. Three nodulation groups were observed on *M. pudica* as described in Table S4 (all nodules being indeterminate): small inefficient nodules (BSa, BN, Figure S2A), small round shape nodules (BSP1, BD, BPL, BSP4, Figure S2B), and elongated nodules with a peanut shape (BSa, BSP3, Figure S2C).

We also incorporated in our analyses strains BR9001 to BR9004, and BR4812 that were isolated from the Piptadenia Group [46] but which were not previously characterized at the taxonomic level, as well as strains from other studies: two from *P. flava* (UPRM8020 & UPRM8021) [42], two from *P. viridiflora* (JPY570, JPY565, Gross *et al*., unpublished), one each from *P. stipulacea* (JPY584), *Pp. blanchetti* (EG100) and *Ps. bahiana* (EG118) (Gross *et al*., unpublished), and three frequent genotypes of *Burkholderia* and *Rhizobium* from a study of *Pp. rigida* symbionts in Uruguay [44]. Based on their 16 S rDNA sequence haplotype, the bacterial collection could be grouped into three genera, with 107 strains in the genus *Burkholderia*, 48 in *Rhizobium* and 41 in *Bradyrhizobium*.

In order to establish if patterns between the occurrence of particular symbionts and plant phylogeny existed, we mapped the % of α- and β-rhizobia (as means across all sampling locations) per legume species onto the *trnL-trnF matK-trnK* phylogeny (Figure 1B). Although some legume species were poorly sampled, *Burkholderia* symbionts were still found along all the phylogeny of the Piptadenia Group species, and in the majority of most sampled species. A few legume species were devoid of *Burkholderia* symbionts, but these were mainly poorly sampled species, and so the presence of β-rhizobia in these species cannot be excluded.

### Taxonomic Characterization of the Piptadenia Symbionts Collection

To further characterize the rhizobial collection at the taxonomic level and assign a putative species to each strain, a phylogenetic analysis of all representative strains based on a partition of two neutral markers (643 bp of the variable part of the 16 S rRNA gene, and a 644 bp fragment of the *recA* gene), was built following a Bayesian analysis (see Mat&Methods). The *recA* gene is a more highly resolved phylogenetic marker than the 16 S rDNA gene, and has proven to be a valuable tool for discriminating species within the *Burkholderia* genus [11], [12], [59]. We also included type strains of the most closely related species in the genera *Burkholderia*, *Rhizobium* and *Bradyrhizobium*. A phylogenetic tree of the 16 S-recA dataset is presented in Figure 2A (only for the genus *Burkholderia*, as α-rhizobia are presented in Figure S3); and individual gene marker phylogenies are available as Figure S4. The bacterial collection was clustered into 9 highly supported clades (posterior probabilities >0.9, bootstrap values >75%) in the genus *Burkholderia* (107 strains), 8 clades in *Rhizobium* (48 strains) and 4 in *Bradyrhizobium* (41 strains). In *Burkholderia*, most strains belonged to clades associated with the species *B. nodosa* (BN, 31 strains), *B. phenoliruptrix* (BPL, 27 strains), *B. sabiae* (BSa, 21 strains), and *B. diazotrophica* (BD, 18 strains). Other strains belonged to *B. phymatum* (BP, one strain), *B. caribensis* (BC, two strains), and three potentially undescribed species: *Burkholderia* sp. 1, 3 and 5 (BSP1, two strains, BSP3, four strains, BSP4, one strain). *Burkholderia* strains were identified among all legume species in this study, except for *Mi. foetidus*, *Ps. psilostachya* and *P. adiantoides*. For these three species, few rhizobial isolates were obtained (3–7), all belonging to *Rhizobium* and/or *Bradyrhizobium* genera. Each clade includes strains isolated from various hosts from the Piptadenia Group, except the BSP3 clade which was sampled only from *P. gonoacantha*. Some clades (BSa, BP, BC, BD, BN) contained strains isolated both from the genus *Mimosa* and the Piptadenia Group, while others were only found associated to the Piptadenia Group (BPL, BSP1, BSP3, BSP4) or to *Mimosa* species (BM, BSy, BSP2, BT). In the case of the α-rhizobia (Figure S3), strains were clustered into 8 new clades of *Rhizobium* and 4 of *Bradyrhizobium*. *Rhizobium* sp. 1 (RSP1), *R. tropici* and RSP3 were the most represented clades with 25, 10 and 8 strains, respectively. *R. tropici* strains were found on the most important number of legume host (6 species). Strains of *R. leucaenae* and *R. gallicum* were also found on *A. peregrina* and *P. flava* [42], respectively. *Rhizobium* isolates from *Pp. rigida* from the Taulé *et al*. study [44] were grouped within the *R. mesoamericanum* species (UYPR7.63 & STM3625; Figure S4), a frequently sampled symbiotic species from *Mimosa pudica* [12], [60], [61]. In *Bradyrhizobium*, four clades could be distinguished and these were named *Bradyrhizobium* sp1 (BrSP1) to BrSP4, with no close relationship to any described species (except BrSP4 that was closely related to *Br. elkanii*). No obvious pattern could be deduced between host plants and α-rhizobial clades. Strains trapped from two soils by *Stryphnodendron* sp. were only characterized on the basis of their 16 S rDNA, and these grouped closed to *Bradyrhizobium* sp. 1 (Figure S4).

### Distribution of Rhizobial Species per Host Plant and Sampling Locations

**Figure 3:**
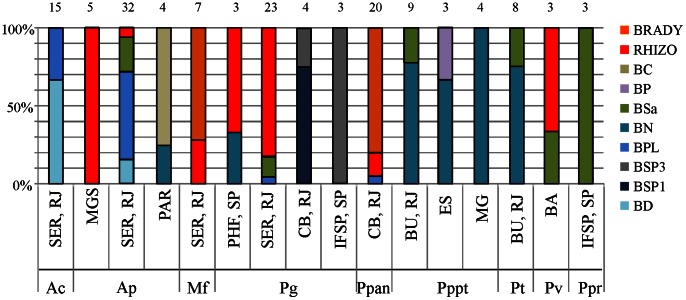
Distribution of rhizobial species according to host plant and sampling regions. The % of each species per legume host was calculated according to sampled isolates listed in Table 1. Sampling region is listed below each histogram while on top are indicated the number of isolates. Abbreviations: SER, RJ: Seropédica, Rio de Janeiro; MGS: Mato Grosso do Sul; PAR: Paranà; SP: Sao Paulo; CB, RJ: Cabo Frio, Rio de Janeiro; BU, RJ: Buzios, Rio de Janeiro; ES: Espirito Santo; MG: Minas Geraïs; PHF, SP: Paraibuna horto florestal, Sao Paulo; BA: Xique-Xique, Bahia; IFSP, SP: IFSP, Sao Paulo; Ac: *Anadenanthera colubrina*, Ap: *A. peregrina*, Mf: *Microlobius foetidus*; Pg: *Piptadenia gonoacantha*; Ppan: *P. paniculata*; Pt: *P. trisperma*; Pv: *P. viridiflora*; Pppt: *Parapiptadenia pterosperma*; Ppr: *Parapiptadenia rigida*.

A distribution of rhizobial species according to their plant host and site of sampling is presented in Figure 3. We observed a strong site sampling effect since the diversity pattern of encountered species was completely divergent for the same host sampled on different sites. This pattern heterogeneity of symbionts was even found between α and β-rhizobia, since *P. gonoacantha* and *A. peregrina* nodules hosted mainly *Rhizobium* strains in some sites but *Burkholderia* species on other sites, indicating no strict specificity of these species for α or β-rhizobia. *Burkholderia nodosa* was particularly frequent on the three different sampling sites of *Pp. pterosperma*, and was also found in *P. trisperma*, *A. peregrina* and *P. gonoacantha*. *Burkholderia sabiae* was also a promiscuous species in the Piptadenia Group (found in *A. peregrina*, *P. viridiflora*, *P. trisperma*, *A. peregrina*). On the other hand, BSP1 and BSP3 species were only found in *P. gonoacantha* nodules. On the plant side, some species exhibited the same pattern of symbionts diversity in the same geographical area (for example *P. trisperma* and *Pp. pterosperma* in Cabo Frio, RJ; Figure 3). However, the opposite could also be found, as *P. gonoacantha* and *P. paniculata* in Cabo Frio also exhibited divergent patterns of symbiotic association, one being nodulated by β- and the other by α-rhizobial species.

### Phylogeny of Symbiosis-related Genes of Piptadenia’s Rhizobia

Phylogenetic analyses were performed on fragments of the *nodC* (involved in the synthesis of the Nod factor core) and *nifH* (involved in nitrogen fixation) genes to evaluate the origin and evolution of symbiosis in Piptadenia Group microsymbionts. The *nodC* and *nifH* data sets used for phylogenies included representative strains listed in Table 1, as well as reference strains from each clade of the Bontemps *et al.* [11] and Mishra *et al*. [12] diversity studies on *Mimosa* species. A *nodC* ML phylogeny is shown in Figure 2B, while the *nifH* ML tree is presented as Figure S5. All *nodC* sequences from *Burkholderia* symbiotic species of the Mimoseae tribe were monophyletic compared to α-rhizobia. *Burkholderia* strains from the Piptadenia Group were clustered into five *nodC* clades (nodC1 to nodC5 on Figure 2B). Some *nodC* clades corresponded to already described bacterial species, such as *B. nodosa* (nodC5), BSP3 (nodC2), while other species had strains carrying different alleles of *nodC* (*e.g. B. sabiae* with nodC1 or nodC3 alleles; *B. phenoliruptrix* with nodC3 and nodC4 alleles). In the case of *B. phenoliruptrix*, strains carrying either nodC3 or nodC4 were both efficient symbionts of *M. pudica,* while *B. sabiae nodC* variants (nodC1, nodC3) exhibited different nodulation phenotypes on *M. pudica* (inefficient/efficient, Table S4). Some clades carried strains sampled from *Mimosa* and Piptadenia Group species (nodC3, C5), while others were specific to either the Piptadenia Group (nodC1, C2, C4) or to *Mimosa* spp. (nodC6, C7). The large nodC3 clade could be divided into 2 sub-clades corresponding to different species: nodC3-1 hosted *B. sabiae* and BSP1, while nodC3-2 contained *B. phymatum*, *B. phenoliruptrix* and *B. diazotrophica* strains. The large nodC3 clade hosted strains from every studied legume species, except for *P. rigida* that was restricted to the nodC1 clade. Overall, no clear pattern was identified concerning *nodC* cladogenesis and legume hosts (except the nodC2 clade that was specific to *P. gonoacantha*). A better correspondence was found between the *nodC* and 16 S-*recA* trees. However, although several 16 S-*recA* clades corresponded to *nodC* clades, horizontal gene transfer was also identified in species which carried different *nodC* alleles (*e.g. B. sabiae* with BSa-1 and 2, and *B. phenoliruptrix* with BPL-1 & 2, Figure 2B). The *nifH* gene ML tree (Figure S5) reflected the same cladogenesis as the *nodC* tree, but with a few exceptions *e.g.* some strains in the nodC1 clade (UYPR1.313, UYPR3.611 and Prigida2) were clustered in a different clade in the *nifH* tree (nifH3).

In the case of α-rhizobia from the Piptadenia Group (Figure S2B), *nodC* sequences from *Rhizobium* strains (*R. tropici*, RSP1, RSP2, *R. leucaenae*) were clustered in a clade (nodC12) together with *R. tropici* strains from diverse *Mimosa* species isolated from Papua New Guinea (NGR181) and Puerto Rico (UPRM8021) [28], [42]. There was no sequence variation on *nodC* fragment between UPRM8021 and the bean symbiont *R. tropici* CIAT899^T^ (not shown). The only exceptions were *R. gallicum* strains from *Piptadenia flava* (*e.g.* UPRM8060) that grouped close to the *R. etli* symbiovar (sv) *mimoseae* clade (nodC8), and to a *R*. *mesoamericanum* strain from *Pp. rigida* (nodC13) that grouped close to *S. meliloti* 1021. The *nodC* and/or *nifH* genes from some *Rhizobium* strains isolated from nodules of *P. paniculata* (STM7339, STM7342) could not be amplified: these strains did not nodulate *M. pudica*, siratro or *P. gonoacantha*, and could not be tested on their original host *P. paniculata*. The symbiotic nature of these two strains thus remains uncertain. *Bradyrhizobium* strains from *P. paniculata* and *Mi. foetidus* clustered in three different clades (nodC9 to nodC11), with the *P. paniculata* isolates (nodC9, C11) being separated from *Mi. foetidus* ones (nodC10). Notably, *Bradyrhizobium* isolates from *P. paniculata* could not be tested on their original host, but they did nodulate siratro but not *M. pudica*. As observed for *Burkholderia* symbionts, the *nifH* tree (Figure S5) reflected the *nodC* groupings of the α-rhizobia.

### Assessment of Symbiotic Specificity in the Piptadenia Group Using Competition Assays

In order to assess plant specificity in the Piptadenia Group, we carried out a coinoculation experiment with 12 rhizobial genotypes (6 *Burkholderia*, 3 *Rhizobium* and 3 *Bradyrhizobium* strains, representative of species and *nodC* clades) on 6 Piptadenia Group species (Mf, Pm, Ppr, Pg, Ap, Ac) for which seeds were available. The final range of nodules analyzed were 12 to 60 on 5 to 9 plant replicates. The nodule occupants were then identified as described in the Material and Methods section. Table 2 shows the percentage of nodule occupancy per bacterial genotype for each plant host. The percentage for α or β-rhizobia was also included in Figure 1C for comparison with wild nodule sampling % and plant phylogeny. Overall, all plant species showed a preference for *Burkholderia* genotypes, except for *Mi. foetidus* which was nodulated preferentially by bradyrhizobia. One *Burkholderia* genotype, STM7353 (BN, nodC5) was the most prevalent (in 38 to 85% of nodules) over all other genotypes on all plant hosts (except for Mf, 9% of nodules), followed by STM7317 (BPL, nodC4) that occupied 30 to 33% of plant hosts (except on Mf, 9%, and Pg, 3%). Interestingly, the two *B. sabiae* genotypes tested (each carrying either the nodC1 or nodC3 allele) did not show the same nodulation pattern: STM7373 (nodC1) was present at around 20% on Mf and Ac while STM7315 (nodC3) was absent, and conversely STM7315 was present at 20% on Pm while STM7373 was absent. Such results indicate potential host plant specificity towards several *nodC* alleles, although competitiveness for nodulation also interferes with these results. Genotypes from the *Rhizobium* and *Bradyrhizobium* genera were poorly competitive, and only STM7379 (RT, nodC2) and STM7378 (BrSP4) were identified in one host, *Mi. foetidus* (these two strains originating from nodules of this species). It is interesting to note that if *Mi. foetidus* favors α-rhizobia symbionts, this plant species selected its native genotypes rather than α-rhizobia from *P. paniculata*, thus underlining specific affinities between this host and its symbionts. Another case of potential host plant specificity is that of STM7296 (BSP3, nodC2), as this poorly competitive strain was only detected on its original plant host, *P. gonoacantha*.

**Table 2:** Nodule occupancy (%) in a coinoculation experiment on 6 species of the Piptadenia group.

Strain&	Species	*nodC* %	Mf	Pm	Ppr	Pg	Ap	Ac
*Burkholderia*								
STM7317 (Pg)	BPL	C4	9	32	33	3	32	31
STM7296 (Pg)	BSP3	C2				7		
STM7399 (Ap)	BD	C3.2					2	
STM7373 (Pppt)	BSa	C1	22	5		3	3	27
STM7315 (Pg)	BSa	C3.1		21	8	2	8	4
STM7353 (Pt)	BN	C5	9	42	59	85	53	38
α-rhizobia								
STM7300 (Pg)	RSP1	C12						
STM7379 (Mf)	RT	C12	17				2	
STM7342 (Ppan)	RSP3	NT						
STM7332 (Ppan)	BrSP1	C11						
STM7329 (Ppan)	BrSP3	C9						
STM7378 (Mf)	BrSP4	NT	43					
Total Nb nodules£			23	19	12	60	60	26

& Original host of each strain is indicated between parenthesis;

% *nodC* numbering corresponds to *nodC* clades defined in Figure 2B and figure S3B;

£ Number of npone.0063478.g005.tifodules analysed per coinoculation assay. Abbreviations: BPL: *Burkholderia phenoliruptrix*; BD: *B. diazotrophica*; BSa: *B. sabiae*; BSP3: *Burkholderia* sp. 3, BN: *B. nodosa*, RSP1,3: *Rhizobium* sp. 1, 3; RT: *Rhizobium tropici*; BrSP1 to 4: *Bradyrhizobium* 1 to 4; Ac: *Anadenanthera colubrina*, Ap: *A. peregrina*, Mf: *Microlobius foetidus*; Pg: *Piptadenia gonoacantha*; Ppr: *Parapiptadenia rigida*; Pm: *Pityrocarpa monoliformis.*

## Discussion

### Burkholderia Species are Common and Highly Diverse Symbionts of the Piptadenia Group

*Burkholderia* symbiont diversity has been intensively studied on collections isolated from nodules of different species of *Mimosa*, most of them originating from Brazil [11], [14]. In the present study we extended the nodulation by *Burkholderia* symbionts to other species in the Piptadenia Group, including six genera that are sister clades to *Mimosa* in the tribe Mimoseae. The Piptadenia Group contains about 70 species (if excluding the genus *Mimosa*, that is also part of this group), which are common in tropical and subtropical America, but are mainly native to Brazil [30]. The capacity of nodulation among these clades has been investigated in previous works (see Introduction), but one of them, *P. trisperma*, is a new report of nodulation from this study. A large diversity of *Burkholderia* was found, with nine species, five being already described as symbionts of *Mimosa* species (*B. sabiae, B. phymatum, B. caribensis, B. diazotrophica, B. nodosa*; [8], [18], [19], [17], [21]), one was not previously reported as containing symbiotic strains (*B. phenoliruptrix*, [10], [62]), and BSP1, BSP3 and BSP4 are three putative new species, whose taxonomic status is currently being investigated (Bournaud *et al*., unpublished). This diversity of symbiotic *Burkholderia* species mirrored the one discovered on 47 species of *Mimosa* by Bontemps *et al* [11] in Brazil, which also had seven clades, of which only three overlap with this study and are symbionts of both *Mimosa* and *Piptadenia* (*B. nodosa, B. diazotrophica* and *B. sabiae*). Overall, diversity studies on symbionts of mimosoid legumes reveal that four rhizobial species were found only associated to the Piptadenia Group (BPL, BSP1, BSP3, BSP4) while four different ones are found only on *Mimosa* species (BSP2, *B. symbiotica, B. mimosarum, B. tuberum*). *B. tuberum* is a frequent species in *Mimosa* spp. nodules in Brazil and French Guiana [11], [12] but was not detected in Piptadenia group nodules. However, these patterns are only based on our current knowledge, and given the high sampling site effect observed on the diversity of symbionts in this group, it is likely that each bacterial species could also be found in both groups of legume hosts.

The legume ability to form a symbiosis with *Burkholderia* is not antagonistic with the presence of α-rhizobia, since the two kinds of symbionts were found on six plant species (Table 1, Figure 1B), and some species had only α-rhizobia strains sampled from them. No clear conclusion about affinities of plant species towards α or β-rhizobia could be drawn for several legumes for which the nodule sampling was limited (*i.e. Py. monoliformis*, *P. flava, P. stipulacea, Pseudopiptadenia* spp), or for which the site sampling effect was too strong (Fig. 3, *P. gonoacantha*, *A. peregrina*). In order to investigate this affinity, we conducted coinoculation tests with 12 genotypes of α and β-rhizobia, and could conclude that *Py. monoliformis*, *Pp. rigida*, *P. gonoacantha*, *A. peregrina* and *A. colubrina* had a strong preference towards *Burkholderia* genotypes, while *Mi. foetidus* had an affinity for two genotypes of *Rhizobium* and *Bradyrhizobium*, as was also found in the wild sampling study. Another species, *P. paniculata*, remains ambiguous in terms of symbiont preference. Unfortunately we could not investigate this topic due to the unavailability of seeds. In the case of *Stryphnodendron* species, trapping assays on soils recovered from the rhizosphere of two trees (identified as *St. adstringens*) trapped only *Bradyrhizobium* species. The species *St. pulcherrimum* species is nodulated by strains close to *R. tropici* but also by *B. nodosa* in French Guiana (Christine Le Roux, unpublished data), indicating that this genus is not only nodulated by α-rhizobia. The *Stryphnodendron* genus thus warrants a more focused study in order to characterize its symbionts.

### Symbiosis Genes in Burkholderia from Mimosoid Legumes are Monophyletic and have been Transferred both Vertically and Horizontally Among Bacterial Species

The *Burkholderia* strains from *Mimosa* and from the other Piptadenia Group species are monophyletic in terms of their *nodC* and *nifH* phylogenies, indicating a common ancestor at the origin of their interaction with members of the tribe Mimoseae. The comparison of symbiotic and neutral markers revealed that vertical gene transfer is the main process for the dissemination of symbiotic genes within *Burkholderia* species. This pattern has been already observed for *Mimosa* symbionts [11], [12], and has allowed for the description of the symbiotic nature of *Mimosa*-nodulating burkholderias as being “ancient”, as it has been tentatively dated at c. 50 MY ago [11]. However, we found also that two species of *Burkholderia* (*B. phenoliruptrix* and *B.sabiae*) harbor strains with different *nodC* alleles, which were either inherited by vertical transfer (nodC3/BSa-2, nodC4/BPL-2), or were acquired by horizontal gene transfer from another *Burkholderia* clade (nodC1/BSa-1, nodC3/BPL-1). Horizontal gene transfer thus also played a role in the co-adaptation between *Burkholderia* species and their legume hosts.

### Host Specificity between Burkholderia Genotypes and Piptadenia Group Species is not Strong

Although gene phylogenies have informed us about the ancient character of the symbiosis between burkholderias and Piptadenias, host specificity between the different partners is not obvious. Host plants are dispersed all over the *Burkholderia* phylogenies (Figure 2A,B), with no specificity pattern, except for that between BSP3 and *P. gonoacantha*. We tried to assess the specificity of the interactions between legume and rhizobial partners using co-inoculation experiments, and detected i) a high affinity of most tested plants towards *Burkholderia* genotypes, ii) promiscuous strains with high competitivity for nodulation (*B. nodosa*, *B. phenoliruptrix*), as well as some trends towards specificity (*e.g. P. gonoacantha/*BSP3, *Mi. foetidus/Bradyrhizobium*). Given the strong site sampling effect detected in the sampling analyses (Figure 3), and the absence of strict host specificity, it is realistic to hypothesize that most Piptadenia Group and *Burkholderia* species interact with a relaxed host specificity, and that plant selection targets nodulation genes from the *Burkholderia nodC* monophyletic clade or from diverse α-rhizobia when no symbiotic *Burkholderia* are present, and there are few exclusions in order to maximize their chances of finding a compatible partner. The environmental (especially soil) conditions would thus play a crucial role in the survival and biogeography of the symbionts, and could be the origin of the different diversity patterns observed in our results. Several parameters have already been found to affect *Burkholderia* symbionts diversity and/or competition, such as soil pH [12], [24], [16], altitude [11], or nitrogen sources [28].

## Conclusion

In this study we extended the symbiotic interaction with *Burkholderia* from *Mimosa* to the wider Piptadenia Group in the tribe Mimoseae. Given the ancient and diverse character of this interaction, symbiosis with *Burkholderia* species might be present in other genera of this tribe, and possibly even in other mimosoid tribes, such as the Ingeae, as Barrett and Parker [63] have isolated *Burkholderia* strains from a member of this tribe (*Abarema macradenia*). The fact that *B. phymatum* STM815 is able to nodulate several legumes in the subfamily Mimosoideae (including species in *Leucaena*, *Prosopis*, and *Acacia*) give clues on the possible extent of the host range of these symbionts [7]. Symbiosis with *Burkholderia* could thus be much more common and ecologically significant than anticipated, encompassing several tribes in the Mimosoideae.

Given the relaxed specificity between Piptadenia Group species and their symbionts, and the diversification of *Burkholderia* into many species, larger samplings of each species in relation to soils and environmental parameters are required in order to get a clearer picture of the diversity and phylogeography of β-rhizobia, and how they have co-evolved with species in the Mimosoideae.

## Supporting Information

Figure S1 **Sampling sites of nodules, soil and plant material.**
(PPT)

Figure S2 **Section of** ***M. pudica*** **and** ***P. gonoacantha*** **nodules induced by different** ***Burkholderia*** **species.** Legend: sections (40 micrometer-deep) of *M. pudica* nodules at 21 days post-inoculation (A to C) induced by *B. sabiae* STM7373 (A), *B. phenoliruptrix* STM7317 (B), or *Burkholderia* sp. 3 STM7296 (C). Sections of *P. gonoacantha* nodules at 60 dpi (D to E) induced by *Burkholderia* sp. 3 STM7296 (D) or *Burkholderia* sp. 1 JPY565 (E). Scale bar: 500 micrometer on all but B (1000 µm).
(PPT)

Figure S3 **Phylogenies of neutral and symbiotic markers in alpha-rhizobia from the Piptadenia group.** The phylogeny of neutral markers (A) is based on a 16 S-*recA* partition and was built by a Bayesian analysis described in Figure 2 legend, while the symbiotic gene *nodC* phylogeny (B) was built by Maximum Likelihood with 1000 bootstraps replicates. See Figure 2 legend and Table S3 for sequence accession numbers.
(PPT)

Figure S4 **Comparison of phylogenies of neutral markers in rhizobia from the Piptadenia group.** Phylogenies of 16 S rDNA (A) and *recA* (B) were built by Neighbor Joining from a distance matrix corrected by the Kimura 2 method, and with 1000 bootstraps replicates). See Figure 2 legend for abbreviations and Table S3 for accession numbers.
(PPT)

Figure S5 **Phylogeny of the** ***nifH*** **gene in alpha and beta-rhizobia from the Piptadenia group of legumes.** The phylogeny was built by ML using a GTR model with 1000 bootstraps replicates. See Figure 2 legend for abbreviations and Table S3 for accession numbers.
(PPT)

Table S1 **Sampling sites characteristics and date of nodule collection.** Legend: £ pH range indicated for the region when soil was not harvested for pH determination. ND: Not Determined.
(DOC)

Table S2 **Primers used for PCR amplification and sequencing.**
(DOC)

Table S3 **Accession numbers of strains from this study and reference strains.** Abbreviations: B. : *Burkholderia*, Br : *Bradyrhizobium*, R. : *Rhizobium*, C: *Cupriavidus*, Pseudo.: *Pseudomonas*, %: T at the end of the strain name indicates the type strain of a species.
(DOC)

Table S4 **Nodulation data of representative strains inoculated on** ***Mimosa pudica*** **(at 21 days post-inoculation-dpi) and** ***Piptadenia gonoacantha*** **(60 dpi).** Legend: £: Efficiency of symbiosis was estimated as effective or ineffective according to plant development and presence of leghaemoglobin in nodules. %: Letters refer to Figure S2 pictures showing nodules sections. NT: Not Tested, ND: Not Determined. Scale bar on all nodules pictures is 1000 mm. BPL: *Burkholderia phenoliruptrix*, BSa: *B*. *sabiae*, BSP1-3: *Burkholderia* sp. 1 to 3, BN: *B. nodosa*, BD: *B. diazotrophica*.
(XLSX)
