# Supplementary material for: Burkholderia Species Are the Most Common and Preferred Nodulating Symbionts of the Piptadenia Group (Tribe Mimoseae)
Source: PLoS One. 2013 May 15;8(5):e63478. doi: 10.1371/journal.pone.0063478 (PMC3655174; doi:10.1371/journal.pone.0063478)
Supplement: Figure S1 — Sampling sites of nodules, soil and plant material. (PPT) [file pone.0063478.s001.ppt]

## Slide 1
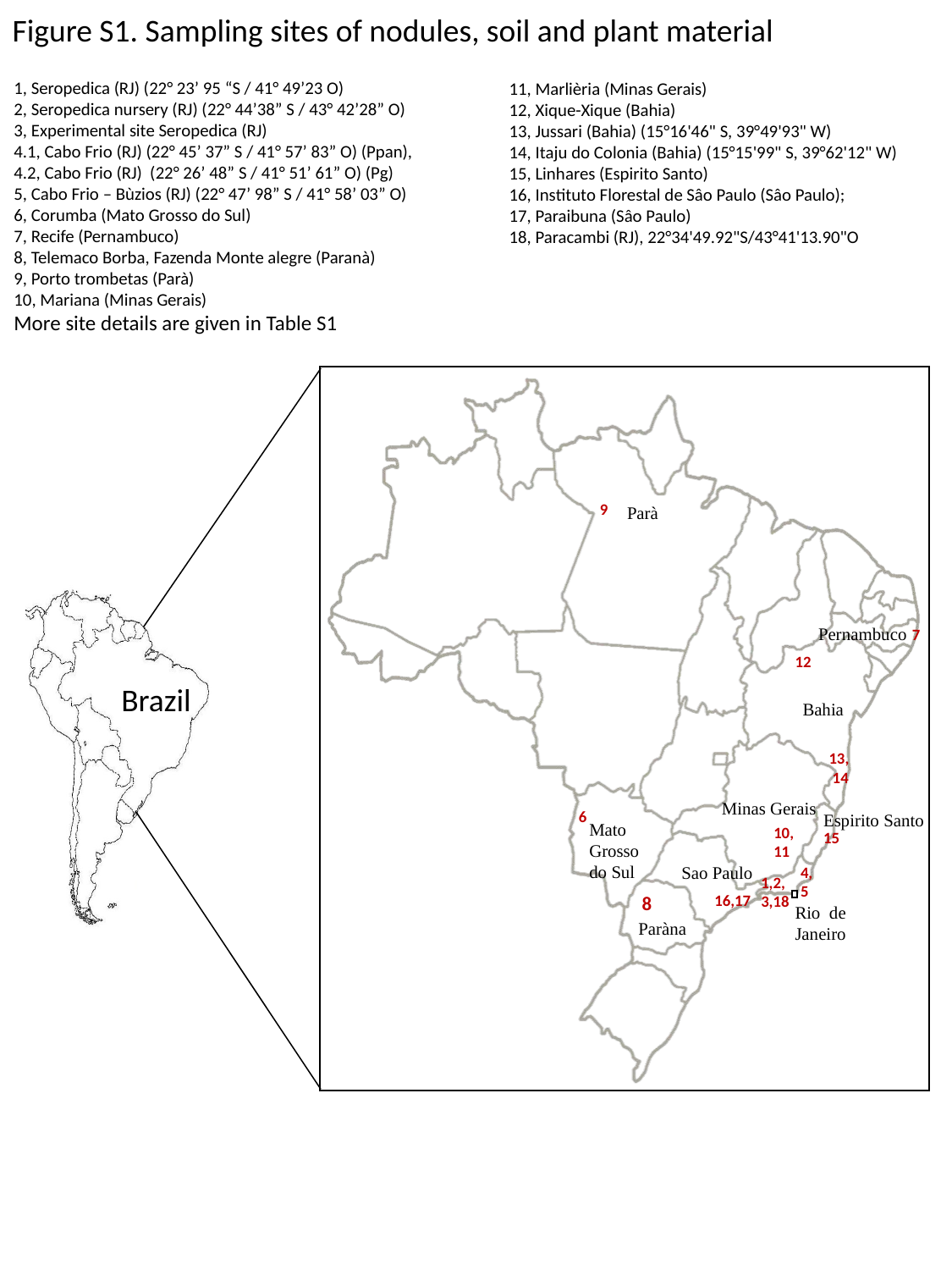

Figure S1. Sampling sites of nodules, soil and plant material
1, Seropedica (RJ) (22° 23’ 95 “S / 41° 49’23 O)
2, Seropedica nursery (RJ) (22° 44’38” S / 43° 42’28” O)
3, Experimental site Seropedica (RJ)
4.1, Cabo Frio (RJ) (22° 45’ 37” S / 41° 57’ 83” O) (Ppan),
4.2, Cabo Frio (RJ) (22° 26’ 48” S / 41° 51’ 61” O) (Pg)
5, Cabo Frio – Bùzios (RJ) (22° 47’ 98” S / 41° 58’ 03” O)
6, Corumba (Mato Grosso do Sul)
7, Recife (Pernambuco)
8, Telemaco Borba, Fazenda Monte alegre (Paranà)
9, Porto trombetas (Parà)
10, Mariana (Minas Gerais)
More site details are given in Table S1
11, Marlièria (Minas Gerais)
12, Xique-Xique (Bahia)
13, Jussari (Bahia) (15°16'46" S, 39°49'93" W)
14, Itaju do Colonia (Bahia) (15°15'99" S, 39°62'12" W)
15, Linhares (Espirito Santo)
16, Instituto Florestal de Sâo Paulo (Sâo Paulo);
17, Paraibuna (Sâo Paulo)
18, Paracambi (RJ), 22°34'49.92"S/43°41'13.90"O
9
Parà
Pernambuco
7
12
Brazil
Bahia
Minas Gerais
6
Espirito Santo
Mato Grosso
do Sul
10, 11
15
Sao Paulo
4,5
1,2, 3,18
16,17
Rio de Janeiro
13,
 14
8
Paràna
