# Supplementary material for: Burkholderia Species Are the Most Common and Preferred Nodulating Symbionts of the Piptadenia Group (Tribe Mimoseae)
Source: PLoS One. 2013 May 15;8(5):e63478. doi: 10.1371/journal.pone.0063478 (PMC3655174; doi:10.1371/journal.pone.0063478)
Supplement: Figure S2 — Section of M. pudica and P. gonoacantha nodules induced by different Burkholderia species. Legend: sections (40 micrometer-deep) of M. pudica nodules at 21 days post-inoculation (A to C) induced by B. sabiae STM7373 (A), B. phenoliruptrix STM7317 (B), or Burkholderia sp. 3 STM7296 (C). Sections of P. gonoacantha nodules at 60 dpi (D to E) induced by Burkholderia sp. 3 STM7296 (D) or Burkholderia sp. 1 JPY565 (E). Scale bar: 500 micrometer on all but B (1000 µm). (PPT) [file pone.0063478.s002.ppt]

## Slide 1
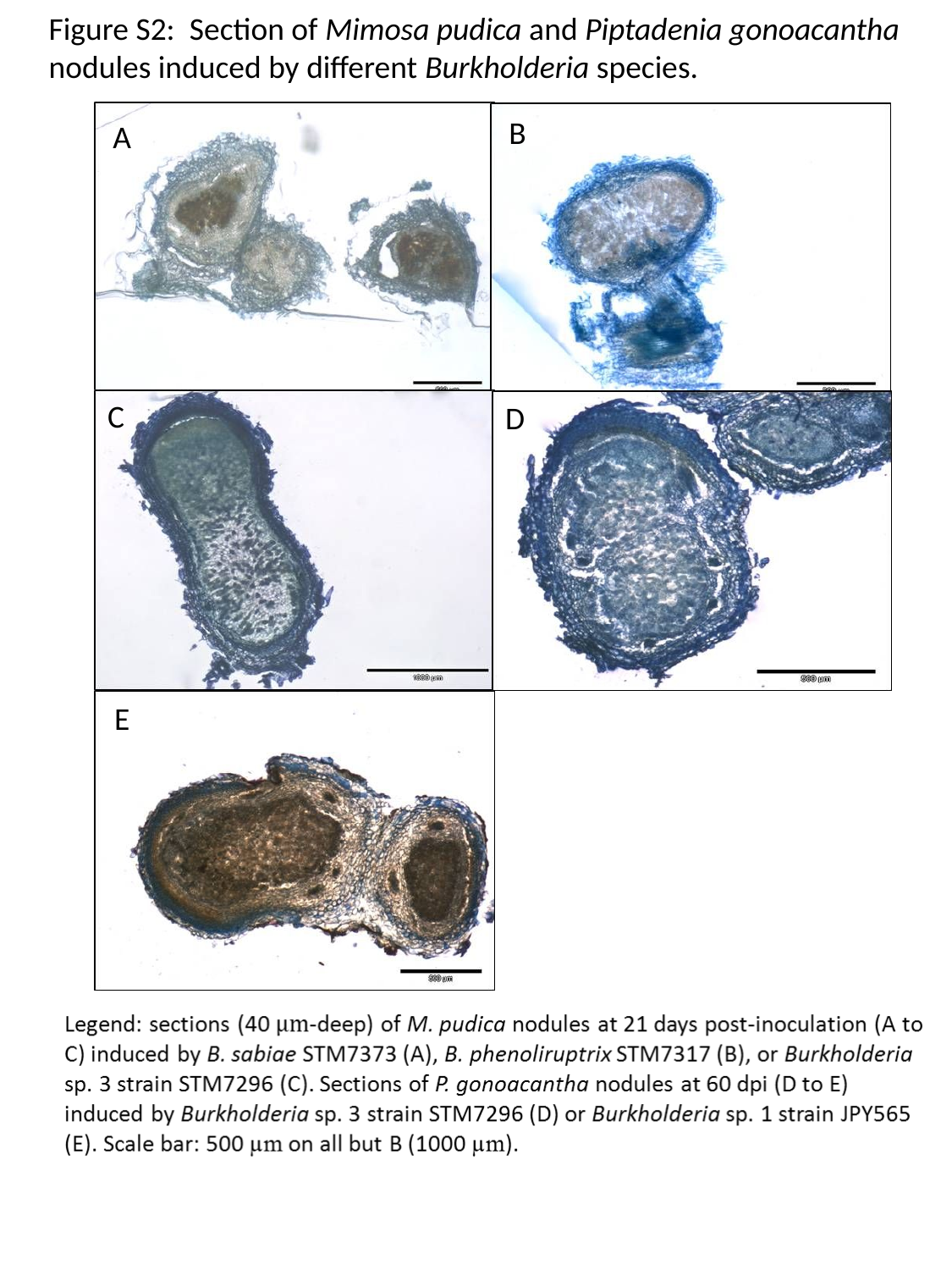

Figure S2: Section of Mimosa pudica and Piptadenia gonoacantha nodules induced by different Burkholderia species.
B
A
C
D
E
