# Supplementary material for: Burkholderia Species Are the Most Common and Preferred Nodulating Symbionts of the Piptadenia Group (Tribe Mimoseae)
Source: PLoS One. 2013 May 15;8(5):e63478. doi: 10.1371/journal.pone.0063478 (PMC3655174; doi:10.1371/journal.pone.0063478)
Supplement: Figure S3 — Phylogenies of neutral and symbiotic markers in alpha-rhizobia from the Piptadenia group. The phylogeny of neutral markers (A) is based on a 16 S-recA partition and was built by a Bayesian analysis described in Figure 2 legend, while the symbiotic gene nodC phylogeny (B) was built by Maximum Likelihood with 1000 bootstraps replicates. See Figure 2 legend and Table S3 for sequence accession numbers. (PPT) [file pone.0063478.s003.ppt]

## Slide 1
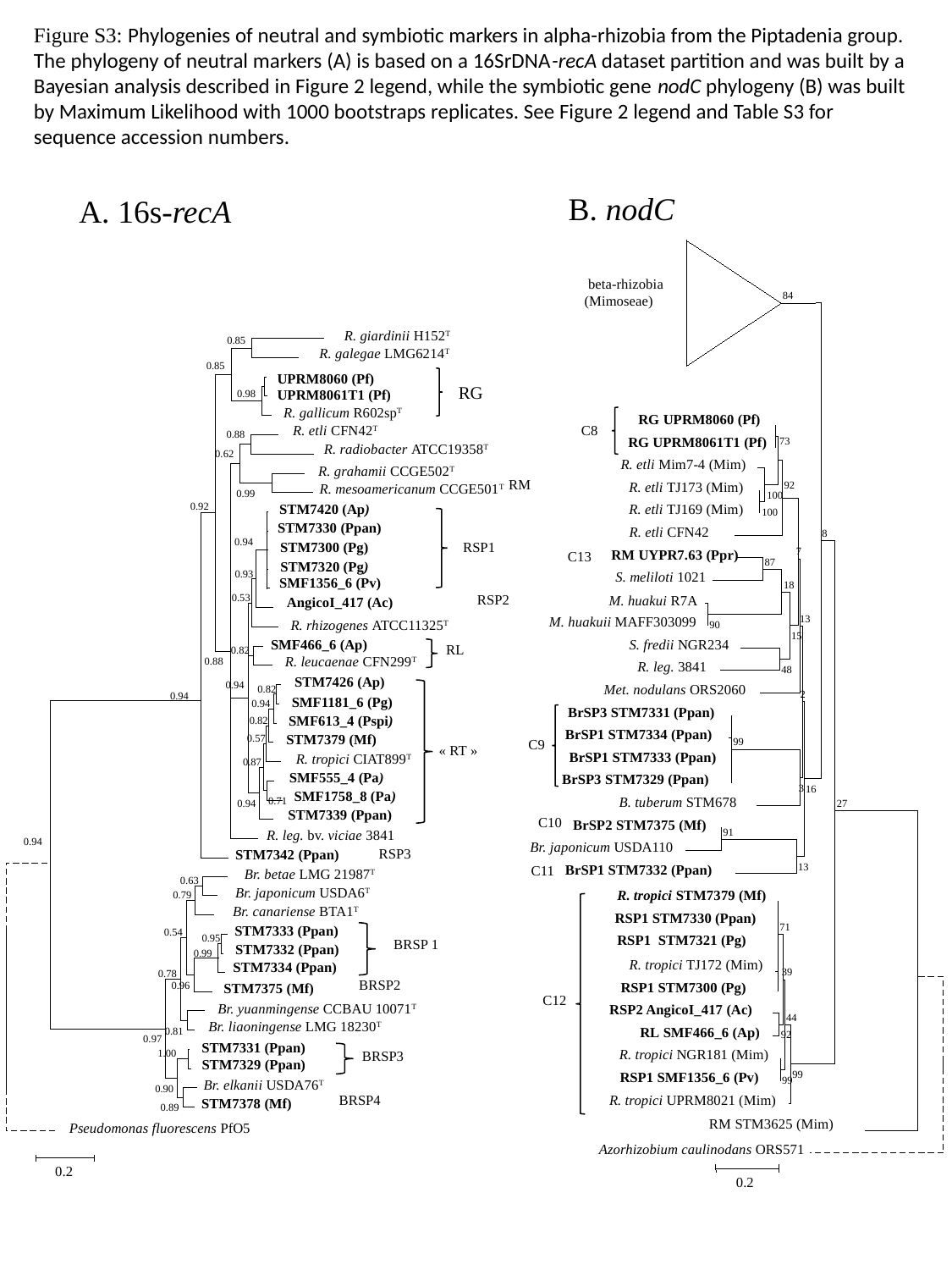

Figure S3: Phylogenies of neutral and symbiotic markers in alpha-rhizobia from the Piptadenia group. The phylogeny of neutral markers (A) is based on a 16SrDNA-recA dataset partition and was built by a Bayesian analysis described in Figure 2 legend, while the symbiotic gene nodC phylogeny (B) was built by Maximum Likelihood with 1000 bootstraps replicates. See Figure 2 legend and Table S3 for sequence accession numbers.
B. nodC
A. 16s-recA
 beta-rhizobia
(Mimoseae)
84
 RG UPRM8060 (Pf)
C8
RG UPRM8061T1 (Pf)
73
 R. etli Mim7-4 (Mim)
92
 R. etli TJ173 (Mim)
100
 R. etli TJ169 (Mim)
100
 R. etli CFN42
8
C13
7
 RM UYPR7.63 (Ppr)
87
 S. meliloti 1021
18
 M. huakui R7A
13
 M. huakuii MAFF303099
90
15
 S. fredii NGR234
 R. leg. 3841
48
Met. nodulans ORS2060
2
 BrSP3 STM7331 (Ppan)
 BrSP1 STM7334 (Ppan)
C9
99
 BrSP1 STM7333 (Ppan)
 BrSP3 STM7329 (Ppan)
3
16
 B. tuberum STM678
27
C10
 BrSP2 STM7375 (Mf)
91
 Br. japonicum USDA110
C11
13
 BrSP1 STM7332 (Ppan)
 R. tropici STM7379 (Mf)
 RSP1 STM7330 (Ppan)
71
RSP1 STM7321 (Pg)
 R. tropici TJ172 (Mim)
39
 RSP1 STM7300 (Pg)
C12
 RSP2 AngicoI_417 (Ac)
44
 RL SMF466_6 (Ap)
92
 R. tropici NGR181 (Mim)
99
RSP1 SMF1356_6 (Pv)
99
 R. tropici UPRM8021 (Mim)
 RM STM3625 (Mim)
 Azorhizobium caulinodans ORS571
0.2
 R. giardinii H152T
0.85
 R. galegae LMG6214T
0.85
UPRM8060 (Pf)
RG
UPRM8061T1 (Pf)
0.98
 R. gallicum R602spT
 R. etli CFN42T
0.88
 R. radiobacter ATCC19358T
0.62
 R. grahamii CCGE502T
RM
 R. mesoamericanum CCGE501T
0.99
0.92
 STM7420 (Ap)
 STM7330 (Ppan)
RSP1
0.94
 STM7300 (Pg)
 STM7320 (Pg)
0.93
 SMF1356_6 (Pv)
RSP2
0.53
 AngicoI_417 (Ac)
 R. rhizogenes ATCC11325T
RL
 SMF466_6 (Ap)
0.82
 R. leucaenae CFN299T
0.88
 STM7426 (Ap)
0.94
0.82
0.94
SMF1181_6 (Pg)
0.94
 SMF613_4 (Pspi)
0.82
 STM7379 (Mf)
0.57
« RT »
 R. tropici CIAT899T
0.87
 SMF555_4 (Pa)
 SMF1758_8 (Pa)
0.71
0.94
 STM7339 (Ppan)
 R. leg. bv. viciae 3841
0.94
RSP3
 STM7342 (Ppan)
 Br. betae LMG 21987T
0.63
 Br. japonicum USDA6T
0.79
 Br. canariense BTA1T
 STM7333 (Ppan)
0.54
BRSP 1
0.95
 STM7332 (Ppan)
0.99
 STM7334 (Ppan)
0.78
BRSP2
0.96
 STM7375 (Mf)
 Br. yuanmingense CCBAU 10071T
 Br. liaoningense LMG 18230T
0.81
0.97
 STM7331 (Ppan)
BRSP3
1.00
 STM7329 (Ppan)
 Br. elkanii USDA76T
0.90
BRSP4
 STM7378 (Mf)
0.89
 Pseudomonas fluorescens PfO5
0.2
