# Supplementary material for: Burkholderia Species Are the Most Common and Preferred Nodulating Symbionts of the Piptadenia Group (Tribe Mimoseae)
Source: PLoS One. 2013 May 15;8(5):e63478. doi: 10.1371/journal.pone.0063478 (PMC3655174; doi:10.1371/journal.pone.0063478)
Supplement: Figure S4 — Comparison of phylogenies of neutral markers in rhizobia from the Piptadenia group. Phylogenies of 16 S rDNA (A) and recA (B) were built by Neighbor Joining from a distance matrix corrected by the Kimura 2 method, and with 1000 bootstraps replicates). See Figure 2 legend for abbreviations and Table S3 for accession numbers. (PPT) [file pone.0063478.s004.ppt]

## Slide 1
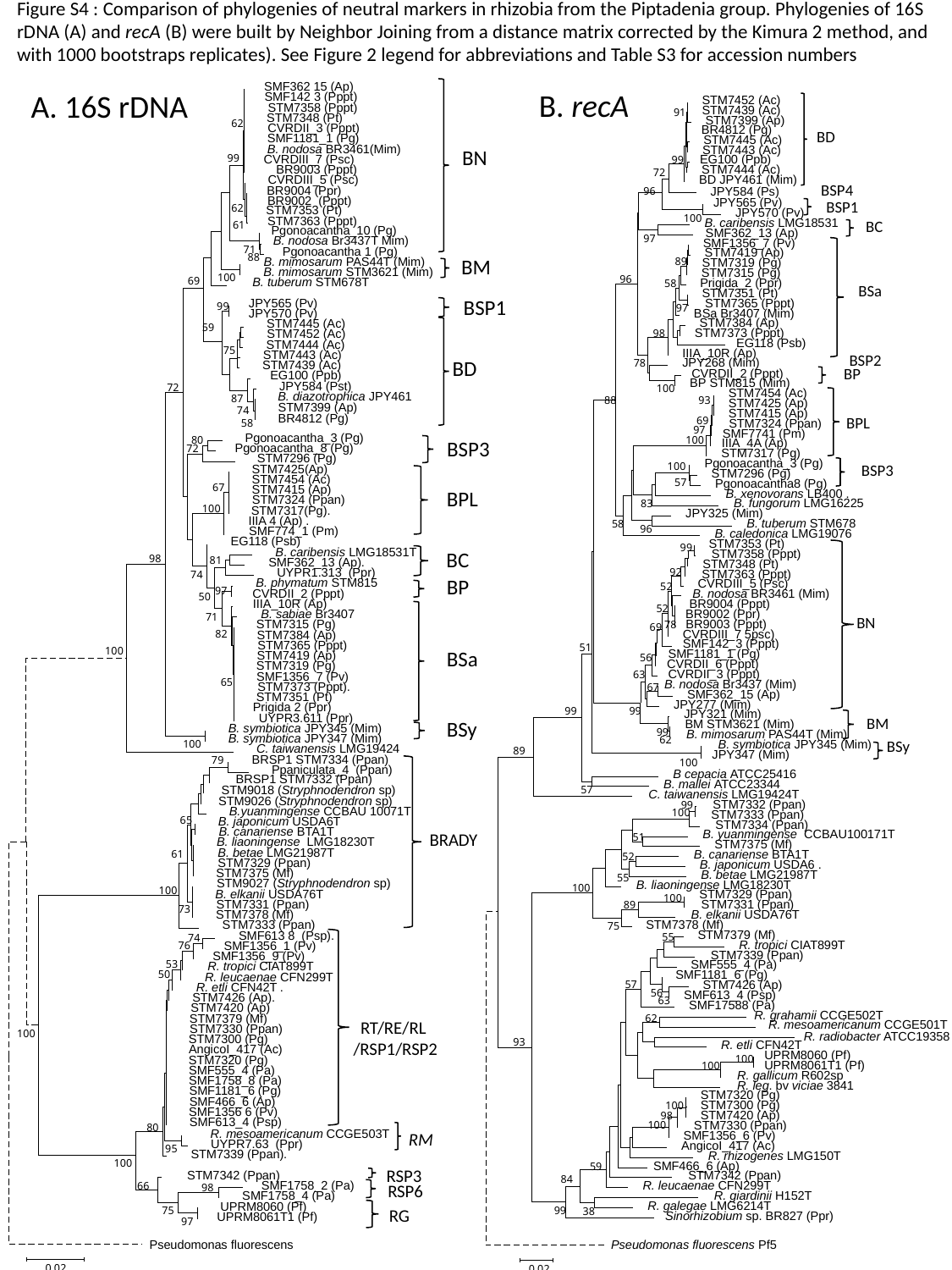

Figure S4 : Comparison of phylogenies of neutral markers in rhizobia from the Piptadenia group. Phylogenies of 16S rDNA (A) and recA (B) were built by Neighbor Joining from a distance matrix corrected by the Kimura 2 method, and with 1000 bootstraps replicates). See Figure 2 legend for abbreviations and Table S3 for accession numbers
SMF362 15 (Ap)
B. recA
A. 16S rDNA
SMF142 3 (Pppt)
62
99
62
61
71
88
100
69
99
59
75
72
87
74
58
80
72
67
100
98
81
74
97
50
71
82
100
65
100
79
65
61
100
73
74
76
53
50
100
80
95
100
66
98
75
97
0.02
 STM7452 (Ac)
 STM7358 (Pppt)
 STM7439 (Ac)
91
 STM7348 (Pt)
 STM7399 (Ap)
BD
 CVRDII_3 (Pppt)
 BR4812 (Pg)
 SMF1181_1 (Pg)
 STM7445 (Ac)
BN
 B. nodosa BR3461(Mim)
 STM7443 (Ac)
CVRDIII_7 (Psc)
EG100 (Ppb)
99
BR9003 (Pppt)
 STM7444 (Ac)
72
 BD JPY461 (Mim)
 CVRDIII_5 (Psc)
BSP4
BR9004 (Ppr)
 JPY584 (Ps)
96
BSP1
BR9002 (Pppt)
JPY565 (Pv)
 STM7353 (Pt)
 JPY570 (Pv)
BC
100
 STM7363 (Pppt)
 B. caribensis LMG18531
 Pgonoacantha_10 (Pg)
SMF362_13 (Ap)
97
 B. nodosa Br3437T Mim)
 SMF1356_7 (Pv)
 Pgonoacantha 1 (Pg)
 STM7419 (Ap)
BM
 B. mimosarum PAS44T (Mim)
89
 STM7319 (Pg)
 B. mimosarum STM3621 (Mim)
 STM7315 (Pg)
96
 B. tuberum STM678T
BSa
 Prigida_2 (Ppr)
58
 STM7351 (Pt)
BSP1
JPY565 (Pv)
 STM7365 (Pppt)
97
 BSa Br3407 (Mim)
JPY570 (Pv)
 STM7384 (Ap)
 STM7445 (Ac)
 STM7373 (Pppt)
 STM7452 (Ac)
98
EG118 (Psb)
 STM7444 (Ac)
BSP2
 IIIA_10R (Ap)
 STM7443 (Ac)
BD
 JPY268 (Mim)
78
 STM7439 (Ac)
BP
CVRDII_2 (Pppt)
EG100 (Ppb)
 BP STM815 (Mim)
 JPY584 (Pst)
100
 STM7454 (Ac)
 B. diazotrophica JPY461
88
93
 STM7425 (Ap)
 STM7399 (Ap)
 STM7415 (Ap)
BPL
 BR4812 (Pg)
69
 STM7324 (Ppan)
97
SMF7741 (Pm)
BSP3
 Pgonoacantha_3 (Pg)
100
IIIA_4A (Ap)
Pgonoacantha_8 (Pg)
 STM7317 (Pg)
 STM7296 (Pg)
BSP3
 Pgonoacantha_3 (Pg)
100
 STM7425(Ap)
 STM7296 (Pg)
 STM7454 (Ac)
 Pgonoacantha8 (Pg)
57
BPL
 STM7415 (Ap)
 B. xenovorans LB400 .
 STM7324 (Ppan)
 B. fungorum LMG16225
83
 STM7317(Pg).
 JPY325 (Mim)
 IIIA 4 (Ap) .
 B. tuberum STM678
58
96
 SMF774_1 (Pm)
 B. caledonica LMG19076
EG118 (Psb)
 STM7353 (Pt)
BC
99
 B. caribensis LMG18531T
 STM7358 (Pppt)
 SMF362_13 (Ap).
 STM7348 (Pt)
 UYPR1.313 (Ppr)
92
 STM7363 (Pppt)
BP
 B. phymatum STM815
 CVRDIII_5 (Psc)
52
CVRDII_2 (Pppt)
B. nodosa BR3461 (Mim)
IIIA_10R (Ap)
 BR9004 (Pppt)
52
 B. sabiae Br3407
BR9002 (Ppr)
BN
BR9003 (Pppt)
 STM7315 (Pg)
78
69
 CVRDIII_7 5psc)
 STM7384 (Ap)
SMF142_3 (Pppt)
 STM7365 (Pppt)
BSa
51
SMF1181_1 (Pg)
 STM7419 (Ap)
56
CVRDII_6 (Pppt)
 STM7319 (Pg)
CVRDII_3 (Pppt)
63
 SMF1356_7 (Pv)
 B. nodosa Br3437 (Mim)
 STM7373 (Pppt).
67
 SMF362_15 (Ap)
 STM7351 (Pt)
 JPY277 (Mim)
Prigida 2 (Ppr)
99
99
 JPY321 (Mim)
BM
BSy
 UYPR3.611 (Ppr)
 BM STM3621 (Mim)
 B. symbiotica JPY345 (Mim)
99
 B. mimosarum PAS44T (Mim)
BSy
 B. symbiotica JPY347 (Mim)
62
 B. symbiotica JPY345 (Mim)
 C. taiwanensis LMG19424
89
JPY347 (Mim)
 BRSP1 STM7334 (Ppan)
100
 Ppaniculata_4 (Ppan)
 B cepacia ATCC25416
 BRSP1 STM7332 (Ppan)
 B. mallei ATCC23344
 STM9018 (Stryphnodendron sp)
57
 C. taiwanensis LMG19424T
 STM9026 (Stryphnodendron sp)
 STM7332 (Ppan)
99
 B.yuanmingense CCBAU 10071T
100
 STM7333 (Ppan)
 B. japonicum USDA6T
 STM7334 (Ppan)
BRADY
 B. canariense BTA1T
 B. yuanmingense CCBAU100171T
51
 B. liaoningense LMG18230T
 STM7375 (Mf)
 B. betae LMG21987T
 B. canariense BTA1T
52
 STM7329 (Ppan)
 B. japonicum USDA6 .
 STM7375 (Mf)
 B. betae LMG21987T
55
 STM9027 (Stryphnodendron sp)
 B. liaoningense LMG18230T
100
 B. elkanii USDA76T
 STM7329 (Ppan)
100
 STM7331 (Ppan)
 STM7331 (Ppan)
89
 STM7378 (Mf)
 B. elkanii USDA76T
 STM7378 (Mf)
 STM7333 (Ppan)
75
 STM7379 (Mf)
 SMF613 8 (Psp).
55
 R. tropici CIAT899T
SMF1356_1 (Pv)
 STM7339 (Ppan)
SMF1356_9 (Pv)
 SMF555_4 (Pa)
 R. tropici CIAT899T
SMF1181_6 (Pg)
 R. leucaenae CFN299T
 STM7426 (Ap)
57
 R. etli CFN42T .
56
 SMF613_4 (Psp)
 STM7426 (Ap).
63
 SMF17588 (Pa)
 STM7420 (Ap)
 R. grahamii CCGE502T
RT/RE/RL
/RSP1/RSP2
 STM7379 (Mf)
62
 R. mesoamericanum CCGE501T
 STM7330 (Ppan)
 R. radiobacter ATCC19358
 STM7300 (Pg)
93
 R. etli CFN42T
 AngicoI_417 (Ac)
UPRM8060 (Pf)
 STM7320 (Pg)
100
UPRM8061T1 (Pf)
100
 SMF555_4 (Pa)
 R. gallicum R602sp
 SMF1758_8 (Pa)
 R. leg. bv viciae 3841
 SMF1181_6 (Pg)
 STM7320 (Pg)
 SMF466_6 (Ap)
 STM7300 (Pg)
100
 SMF1356 6 (Pv)
 STM7420 (Ap)
98
 SMF613_4 (Psp)
 STM7330 (Ppan)
100
RM
 R. mesoamericanum CCGE503T
 SMF1356_6 (Pv)
 UYPR7.63 (Ppr)
 AngicoI_417 (Ac)
 STM7339 (Ppan).
 R. rhizogenes LMG150T
RSP3
 SMF466_6 (Ap)
59
 STM7342 (Ppan)
 STM7342 (Ppan)
84
RSP6
 R. leucaenae CFN299T
SMF1758_2 (Pa)
 R. giardinii H152T
 SMF1758_4 (Pa)
RG
 R. galegae LMG6214T
 UPRM8060 (Pf)
99
38
Sinorhizobium sp. BR827 (Ppr)
UPRM8061T1 (Pf)
Pseudomonas fluorescens
 Pseudomonas fluorescens Pf5
0.02
