# Supplementary material for: Burkholderia Species Are the Most Common and Preferred Nodulating Symbionts of the Piptadenia Group (Tribe Mimoseae)
Source: PLoS One. 2013 May 15;8(5):e63478. doi: 10.1371/journal.pone.0063478 (PMC3655174; doi:10.1371/journal.pone.0063478)
Supplement: Figure S5 — Phylogeny of the nifH gene in alpha and beta-rhizobia from the Piptadenia group of legumes. The phylogeny was built by ML using a GTR model with 1000 bootstraps replicates. See Figure 2 legend for abbreviations and Table S3 for accession numbers. (PPT) [file pone.0063478.s005.ppt]

## Slide 1
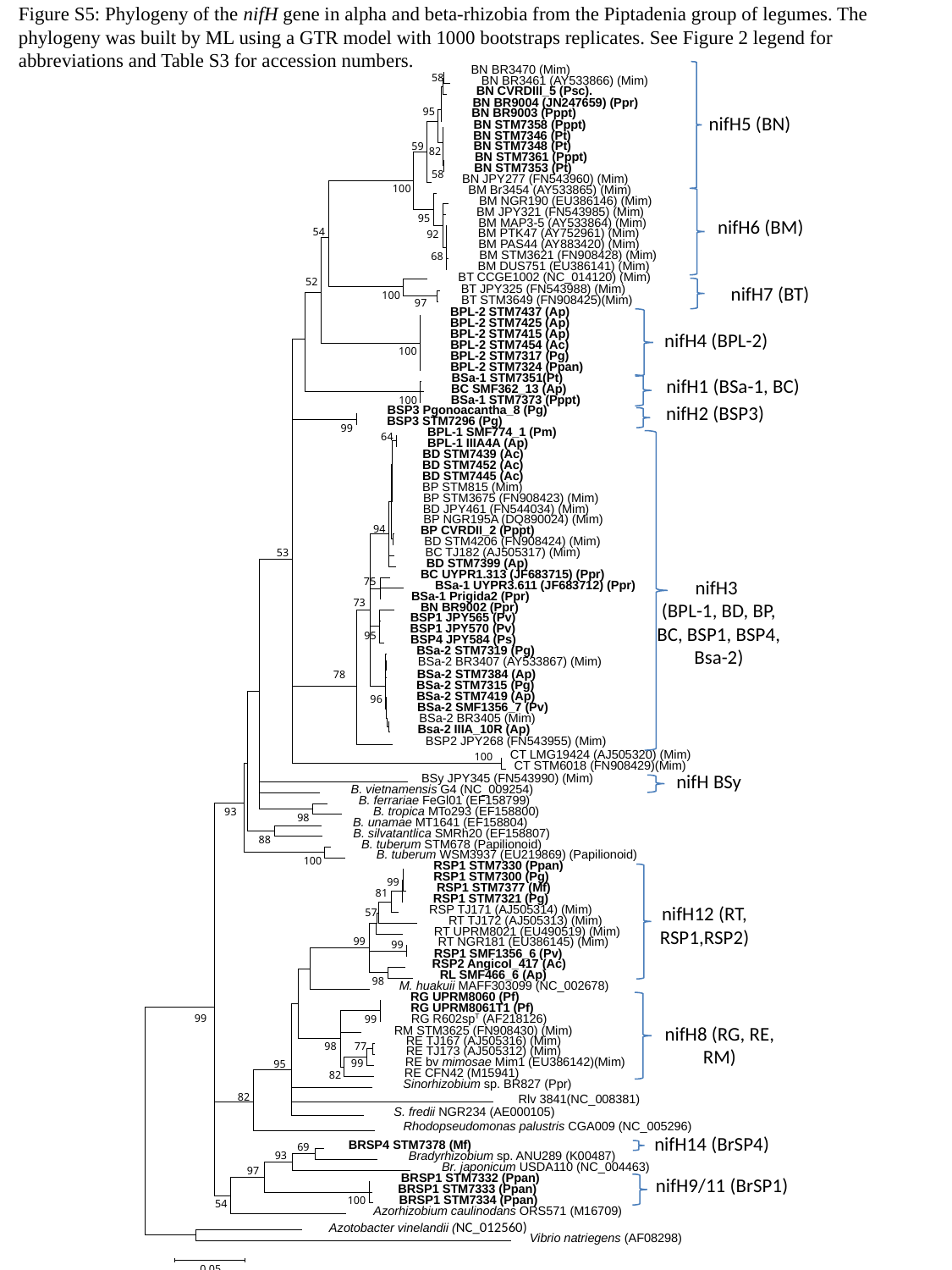

Figure S5: Phylogeny of the nifH gene in alpha and beta-rhizobia from the Piptadenia group of legumes. The phylogeny was built by ML using a GTR model with 1000 bootstraps replicates. See Figure 2 legend for abbreviations and Table S3 for accession numbers.
BN BR3470 (Mim)
58
 BN BR3461 (AY533866) (Mim)
 BN CVRDIII_5 (Psc).
 BN BR9004 (JN247659) (Ppr)
95
nifH5 (BN)
 BN BR9003 (Pppt)
 BN STM7358 (Pppt)
 BN STM7346 (Pt)
 BN STM7348 (Pt)
59
82
 BN STM7361 (Pppt)
 BN STM7353 (Pt)
58
 BN JPY277 (FN543960) (Mim)
 BM Br3454 (AY533865) (Mim)
100
 BM NGR190 (EU386146) (Mim)
 BM JPY321 (FN543985) (Mim)
nifH6 (BM)
95
 BM MAP3-5 (AY533864) (Mim)
54
 BM PTK47 (AY752961) (Mim)
92
 BM PAS44 (AY883420) (Mim)
 BM STM3621 (FN908428) (Mim)
68
 BM DUS751 (EU386141) (Mim)
 BT CCGE1002 (NC_014120) (Mim)
52
nifH7 (BT)
 BT JPY325 (FN543988) (Mim)
100
 BT STM3649 (FN908425)(Mim)
97
 BPL-2 STM7437 (Ap)
 BPL-2 STM7425 (Ap)
nifH4 (BPL-2)
 BPL-2 STM7415 (Ap)
 BPL-2 STM7454 (Ac)
100
 BPL-2 STM7317 (Pg)
 BPL-2 STM7324 (Ppan)
nifH1 (BSa-1, BC)
 BSa-1 STM7351(Pt)
 BC SMF362_13 (Ap)
 BSa-1 STM7373 (Pppt)
100
nifH2 (BSP3)
 BSP3 Pgonoacantha_8 (Pg)
 BSP3 STM7296 (Pg)
99
 BPL-1 SMF774_1 (Pm)
64
 BPL-1 IIIA4A (Ap)
 BD STM7439 (Ac)
 BD STM7452 (Ac)
 BD STM7445 (Ac)
 BP STM815 (Mim)
 BP STM3675 (FN908423) (Mim)
 BD JPY461 (FN544034) (Mim)
 BP NGR195A (DQ890024) (Mim)
94
BP CVRDII_2 (Pppt)
 BD STM4206 (FN908424) (Mim)
 BC TJ182 (AJ505317) (Mim)
53
 BD STM7399 (Ap)
 BC UYPR1.313 (JF683715) (Ppr)
nifH3
(BPL-1, BD, BP, BC, BSP1, BSP4, Bsa-2)
75
 BSa-1 UYPR3.611 (JF683712) (Ppr)
 BSa-1 Prigida2 (Ppr)
73
BN BR9002 (Ppr)
 BSP1 JPY565 (Pv)
 BSP1 JPY570 (Pv)
95
BSP4 JPY584 (Ps)
 BSa-2 STM7319 (Pg)
 BSa-2 BR3407 (AY533867) (Mim)
 BSa-2 STM7384 (Ap)
78
 BSa-2 STM7315 (Pg)
 BSa-2 STM7419 (Ap)
96
 BSa-2 SMF1356_7 (Pv)
 BSa-2 BR3405 (Mim)
Bsa-2 IIIA_10R (Ap)
 BSP2 JPY268 (FN543955) (Mim)
 CT LMG19424 (AJ505320) (Mim)
100
 CT STM6018 (FN908429)(Mim)
nifH BSy
 BSy JPY345 (FN543990) (Mim)
 B. vietnamensis G4 (NC_009254)
 B. ferrariae FeGl01 (EF158799)
 B. tropica MTo293 (EF158800)
93
98
 B. unamae MT1641 (EF158804)
 B. silvatantlica SMRh20 (EF158807)
88
 B. tuberum STM678 (Papilionoid)
 B. tuberum WSM3937 (EU219869) (Papilionoid)
100
 RSP1 STM7330 (Ppan)
 RSP1 STM7300 (Pg)
99
 RSP1 STM7377 (Mf)
81
 RSP1 STM7321 (Pg)
nifH12 (RT, RSP1,RSP2)
 RSP TJ171 (AJ505314) (Mim)
57
 RT TJ172 (AJ505313) (Mim)
 RT UPRM8021 (EU490519) (Mim)
 RT NGR181 (EU386145) (Mim)
99
99
RSP1 SMF1356_6 (Pv)
RSP2 AngicoI_417 (Ac)
RL SMF466_6 (Ap)
98
 M. huakuii MAFF303099 (NC_002678)
 RG UPRM8060 (Pf)
 RG UPRM8061T1 (Pf)
 RG R602spT (AF218126)
99
99
nifH8 (RG, RE, RM)
 RM STM3625 (FN908430) (Mim)
 RE TJ167 (AJ505316) (Mim)
98
77
 RE TJ173 (AJ505312) (Mim)
 RE bv mimosae Mim1 (EU386142)(Mim)
99
95
 RE CFN42 (M15941)
82
 Sinorhizobium sp. BR827 (Ppr)
82
 Rlv 3841(NC_008381)
 S. fredii NGR234 (AE000105)
 Rhodopseudomonas palustris CGA009 (NC_005296)
nifH14 (BrSP4)
BRSP4 STM7378 (Mf)
69
 Bradyrhizobium sp. ANU289 (K00487)
93
 Br. japonicum USDA110 (NC_004463)
97
nifH9/11 (BrSP1)
 BRSP1 STM7332 (Ppan)
 BRSP1 STM7333 (Ppan)
 BRSP1 STM7334 (Ppan)
100
54
 Azorhizobium caulinodans ORS571 (M16709)
 Azotobacter vinelandii (NC_012560)
 Vibrio natriegens (AF08298)
0.05
