# Supplementary material for: Burkholderia Species Are the Most Common and Preferred Nodulating Symbionts of the Piptadenia Group (Tribe Mimoseae)
Source: PLoS One. 2013 May 15;8(5):e63478. doi: 10.1371/journal.pone.0063478 (PMC3655174; doi:10.1371/journal.pone.0063478)
Supplement: Table S1 — Sampling sites characteristics and date of nodule collection. Legend: £ pH range indicated for the region when soil was not harvested for pH determination. ND: Not Determined. (DOC) [file pone.0063478.s006.doc]

| Table S1 : Sampling sites characteristics and date of nodule collection | | | |  |  |  |
| --- | --- | --- | --- | --- | --- | --- |
| Sampling site | Sampled species | Rep. strains | type of soil (brazilian classification) | soil pH^£^ | year | season |
| 1, Seropedica (RJ) (22° 23’ 95 “S / 41° 49’23 O) | Microlobius foetidus | STM7375, 7378, 7379 | Podzolic vermelho amarelo pozolic red yellow | 5.2 | April 2010 | Fall (begin of dry season) |
| 2, Seropedica nursery (RJ) (22° 44’38” S / 43° 42’28” O) | Piptadenia gonoacantha, Anadenanthera peregrina, A. colubrina | STM7321, 7300, 7315, 7319, 7317, 7420, 7426, 7419, 7384, 7399, 7437, 7415, 7444, 7439, 7445, 7443, 7452, 7454 | Nursery bed: 1/3 local soil, 1/3 sand, 1/3 clay | 6.7 | April 2010 | Fall (begin of dry season) |
| 3, Experimental site Seropedica (RJ) | Piptadenia monoliformis | SMF774_1 | Podzolic vermelho amarelo pozolic (red yellow) | ND | november 1994 | Spring |
|  | Piptadenia paniculata | STM7339, 7342, 7330, 7333, 7334, 7331, 7332, 7329, 7324 | Podzolic vermelho amarelo pozolic (red yellow) | 6.2 | april 2010 | Fall (begin of dry season) |
| 4.2, Cabo Frio (RJ) (**22° 26’ 42” S / 41° 51’ 41” O)** (Pg) | Piptadenia gonoacantha | STM7296 | Podzolic vermelho amarelo pozolic (red yellow) | 4.7 | April 2010 | Fall (begin of dry season) |
| 5, Cabo Frio – Bùzios (RJ) (22° 47’ 98” S / 41° 58’ 03” O) | Piptadenia trisperma, Parapiptadenia pterosperma | STM7365, 7373, 7363, 7358, 7351, 7353, 7348 | Podzolic vermelho amarelo pozolic (red yellow) | 5.1 | April 2010 | Fall (begin of dry season) |
| 6, Corumba (Mato Grosso do Sul) | Anadenanthera peregrina | SMF466_6 | Nursery bed: 1/3 local soil, 1/3 sand, 1/3 clay | ND | May 1992 | Fall |
| 7, Recife (Pernambuco) | Anadenanthera colubrina | AngicoI_417 | Nursery bed: 1/3 local soil, 1/3 sand, 1/3 clay | ND | ND | ND |
| 8, Telemaco Borba, Fazenda Monte alegre (Paranà) | Anadenanthera peregrina | SMF362_13, SMF362_15 | Nursery bed: 1/3 local soil, 1/3 sand, 1/3 clay | ND | October 1991 | Spring |
| 9, Porto trombetas (Parà) | Pseudopiptadenia psilostachya | SMF613_4 | Nursery bed: 1/3 local soil, 1/3 sand, 1/3 clay | ND | March 1995 | Summer |
| 10, Mariana (Minas Gerais) | Piptadenia adiantoides | SMF1758_4, SMF1758_8 | Nursery bed: 1/3 local soil, 1/3 sand, 1/3 clay | ND | May 2000 | Winter |
| 11, Marlièria (Minas Gerais) | Parapiptadenia pterosperma | SMF142_3 | Nursery bed: 1/3 local soil, 1/3 sand, 1/3 clay | ND | November 1984 | Spring |
| 12, Xique-Xique (Bahia) | Piptadenia viridiflora | SMF1356_6, SMF1356_7 | Nursery bed: 1/3 local soil, 1/3 sand, 1/3 clay | ND | November 1997 | Spring |
| 13, Jussari (Bahia) (15°16'46" S, 39°49'93" W) | Pseudopiptadenia bahiana | EG118 | Argissolo vermelho-amarelo distrófico (Paleudult), nutrient poor acid soil. | 4-5.6 | December 2010 | Begin of rainy season |
| 14, Itaju do Colonia (Bahia) (15°15'99" S, 39°62'12" W) | Parapiptadenia blanchetti | EG100 | Argissolo vermelho-amarelo distrófico (Paleudult), nutrient poor acid soil. | 4-5.6 | November 2010 | Dry season |
| 15, Linhares (Espirito Santo) | Pseudopiptadenia contorta | CVRDIII_5, CVRDIII_7 | Nursery bed: 1/3 local soil, 1/3 sand, 1/3 clay | ND | July 1982 | winter |
| 16, Instituto Florestal de Sâo Paulo (Sâo Paulo) | Anadenanthera peregrina | IIIA_4A, IIIA_10R, P.gonoacantha1,3,8 | Nursery bed: 1/3 local soil, 1/3 sand, 1/3 clay | ND | April 1996 | Fall |
| 17, Paraibuna (Sâo Paulo) | Piptadenia gonoacantha | SMF1181_1, SMF1181_6 | Nursery bed: 1/3 local soil, 1/3 sand, 1/3 clay | ND | April 1996 | Fall |
| 18, Paracambi (RJ), 22°34'49.92"S/43°41'13.90"O | Stryphnodendron sp. | STM9018, 9026, 9027 | Podzolic vermelho amarelo pozolic (red yellow) | 5.7 | April 2010 | Fall |
| £ pH range indicated for the region when soil was not harvested for pH determination. ND: Not Determined. | | | |  |  |  |
