# Supplementary material for: Burkholderia Species Are the Most Common and Preferred Nodulating Symbionts of the Piptadenia Group (Tribe Mimoseae)
Source: PLoS One. 2013 May 15;8(5):e63478. doi: 10.1371/journal.pone.0063478 (PMC3655174; doi:10.1371/journal.pone.0063478)
Supplement: Table S2 — Primers used for PCR amplification and sequencing. (DOC) [file pone.0063478.s007.doc]

Table S2. Primers used for PCR amplification and sequencing.

| **Primer name** | **Primer sequence (5'-3')** | **Bacterial genus targetted** | **Target region** | **Reference** |
| --- | --- | --- | --- | --- |
| fD1 | GAGAGTTTGATCCTGGCTCAG | Most eubacteria | 16S rRNA | Weisburg et al., 1991 |
| rD1 | AAGGAGGTGATCCAGCC | Most eubacteria | 16S rRNA | Weisburg et al., 1991 |
| 16S-1080r # | GGGACTTAACCCAACATCT | Most eubacteria | 16S rRNA | Sy et al., 2001 |
| TSrecAf | CAACTGC**MY**TGCGTATCGTCGAAGG | *Bradyrhizobium* | *recA* | Stepkowski et al., 2005 |
| TSrecAr | CGGATCTGGTTGATGAAGATCACCATG | *Bradyrhizobium* | *recA* | Stepkowski et al., 2005 |
| recAf | ATCGAGCGGTCGTTCGGCAAGGG | *Rhizobium* | *recA* | Gaunt et al., 2001 |
| recAr | TTGCGCAGCGCCTGGCTCAT | *Rhizobium* | *recA* | Gaunt et al., 2001 |
| BUR1 | GATCGA**R**AAGCAGTTCGGCAA | *Burkholderia* | *recA* | Payne et al., 2005 |
| BUR2 | TTGTCCTTGCCCTG**R**CCGAT | *Burkholderia* | *recA* | Payne et al., 2005 |
| nifHr | GC**R**TA**8**A**BN**GCCATCAT**Y**TC | Proteobacteria | *nifH* | Chen et al., 2003 |
| NifHf | AA**R**GG**N**GG**N**AT**Y**GG**H**AA**R**TC | Proteobacteria | *nifH* | Chen et *al.*, 2003 |
| nifHF | TACGG**N**AA**R**GG**S**GG**N**ATCGGCAA | alpha-rhizobia | *nifH* | Laguerre et al., 2001 |
| nifHI | AGCATGTC**Y**TC**S**AG**Y**TC**N**TCCA | alpha-rhizobia | *nifH* | Laguerre et al., 2001 |
| nodCF | A**Y**GT**H**GT**Y**GA**Y**GACGGTTC | *Rhizobium* | *nodC* | Laguerre et al., 2001 |
| nodCI | CG**Y**GACAGCCA**N**TC**K**CTATTG | *Rhizobium* | *nodC* | Laguerre et al., 2001 |
| nodCBurk2F | ACT**S**ATACT**Y**AACGT**M**GA**Y**TC | *Burkholderia* | *nodC* | Bontemps et al., 2009 |
| nodCBurk2R | G**MR**AA**Y**CC**R**AGAAATCGAAG | *Burkholderia* | *nodC* | Bontemps et al., 2009 |
| nodCBurkPipF | T**R**AT**Y**GA**Y**ATGGAATACTGGC | *Burkholderia (B. phenoliruptrix, B. sabiae, Bsp1 and B. diazotrophica)* | *nodC* | this study |
| nodCBurkPipR | CAGCGGA**Y**AT**M**GTCATTGA | *Burkholderia ( B. phenoliruptrix , B. sabiae, Bsp1 and B. diazotrophica)* | *nodC* | this study |
| NodCfor540 | AT**Y**GA**Y**ATGGA**R**TA**Y**TGGCT | *Bradyrhizobium* | *nodC* | Sarita et al., 2005 |
| NodCrev1160 | CG**H**GACAGCCA**R**TCGCT**R**TTG | *Bradyrhizobium* | *nodC* | Sarita et al., 2005 |

# Internal primer used for the 16S rRNA gene sequencing
