# Supplementary material for: Burkholderia Species Are the Most Common and Preferred Nodulating Symbionts of the Piptadenia Group (Tribe Mimoseae)
Source: PLoS One. 2013 May 15;8(5):e63478. doi: 10.1371/journal.pone.0063478 (PMC3655174; doi:10.1371/journal.pone.0063478)
Supplement: Table S3 — Accession numbers of strains from this study and reference strains. Abbreviations: B. : Burkholderia, Br : Bradyrhizobium, R. : Rhizobium, C: Cupriavidus, Pseudo.: Pseudomonas, %: T at the end of the strain name indicates the type strain of a species. (DOC) [file pone.0063478.s008.doc]

Table S3 : Accession numbers of strains from this study and reference strains.

| Original Host/ Strain | Ribotype% | nodC clade | Bacterial species ^&^ | 16S | *recA* | *nodC* | *nifH* |
| --- | --- | --- | --- | --- | --- | --- | --- |
| ***Piptadenia gonoacantha*** | |  |  |  |  |  |  |
| STM7321 | R1 | C12 | *Rhizobium* sp. 1 | HE983657 | HE983709 | HE983784 | HF536729 |
| STM7300 | R1 | C12 | *Rhizobium* sp. 1 | HE983655 | HE983707 | HE983798 | HF536730 |
| SMR1181_6 | R1 | NT | *R. tropici* | HE983647 | HE983761 | NT | NT |
| STM7315 | B1 | C3 | *B. sabiae* | HE983656 | HE983708 | HE983795 | HF536750 |
| STM7319 | B1 | C3 | *B. sabiae* | HE983677 | HE983729 | HE983805 | HF536751 |
| SMF1181_1 | B2 | NT | *B. nodosa* | HE983638 | HE983752 | NT | NT |
| P. gonoacantha_1 | B3 | NT | *B. nodosa* | HE983695 | NT | NT | NT |
| P. gonoacantha_3 | B4 | NT | *Burkholderia* sp. 3 | HE983637 | HE983751 | NT | NT |
| P. gonoacantha_8 | B5 | C2 | *Burkholderia* sp. 3 | HE983645 | HE983759 | HE983814 | HF536753 |
| STM7296 | B6 | C2 | *Burkholderia* sp. 3 | HE983659 | HE983711 | HE983787 | HF536754 |
| STM7317 | B7 | C4 | *B. phenoliruptrix* | HE983658 | HE983710 | HE983777 | HF536737 |
| BR4812 | B8 | NA | *B. diazotrophica* | AY904782 | HE983770 | NA | NT |
|  |  |  |  |  |  |  |  |
| ***Piptadenia trisperma*** | |  |  |  |  |  |  |
| STM7351 | B1 | C1 | *B. sabiae* | HE983662 | HE983714 | HE983794 | HF536756 |
| STM7353 | B9 | C5 | *B. nodosa* | HE983676 | HE983728 | HF536767 | HF536761 |
| STM7348 | B2 | C5 | *B. nodosa* | HE983661 | HE983713 | HE983789 | HF536758 |
|  |  |  |  |  |  |  |  |
| ***Piptadenia paniculata*** | |  |  |  |  |  |  |
| STM7339 | R2 | NA | *R. tropici* | HE983665 | HE983717 | NA | NT |
| STM7342 | R3 | NA | *Rhizobium* sp. 3 | HE983666 | HE983718 | NA | NT |
| STM7330 | R1 | C12 | *Rhizobium* sp. 1 | HE983667 | HE983719 | HE983797 | HF536731 |
| STM7333 | R4 | C9 | *Bradyrhizobium* sp. 1 | HE983663 | HE983715 | HE983778 | HF536765 |
| STM7334 | R5 | C9 | *Bradyrhizobium* sp. 1 | HE983664 | HE983716 | HE983798 | HF536764 |
| STM7331 | R6 | C9 | *Bradyrhizobium* sp. 3 | HE983668 | HE983720 | HE983782 | NT |
| STM7332 | R7 | C11 | *Bradyrhizobium* sp. 1 | HE983669 | HE983721 | HE983780 | HF536763 |
| STM7329 | R8 | C9 | *Bradyrhizobium* sp. 3 | HE983670 | HE983722 | HE983781 | NT |
| STM7324 | B7 | C4 | *B. phenoliruptrix* | HE983671 | HE983723 | HE983779 | HF536734 |
|  |  |  |  |  |  |  |  |
| ***Piptadenia adiantoides*** | |  |  |  |  |  |  |
| SMF1758_4 | R9 | NT | *Rhizobium* sp. 6 | HE983654 | NT | NT | NT |
| SMF1758_8 | R1 | NT | *R.* *tropici* | HE983651 | HE983765 | NT | NT |
|  |  |  |  |  |  |  |  |
| ***Piptadenia monoliformis*** | |  |  |  |  |  |  |
| SMF774_1 | B10 | C3 | *B. phenoliruptrix* | HE983652 | HE983766 | HE983810 | HF536741 |
|  |  |  |  |  |  |  |  |
| ***Piptadenia viridiflora*** | |  |  |  |  |  |  |
| SMF1356_6 | R1 | C12 | *Rhizobium* sp*.* 1 | HE983636 | HE983750 | HE983806 | HF536732 |
| SMF1356_7 | B1 | C3 | *B. sabiae* | HE983646 | HE983760 | HE983812 | HF536746 |
| JPY570 (CAE9) | B11 | C3 | *Burkholderia* sp. 1 | JN247664 | JN247653 | NT | NT |
| JPY565 (CAE1) | B11 | C3 | *Burkholderia* sp. 1 | JN167599 | JN247651 | JN247650 | NT |
|  |  |  |  |  |  |  |  |
| ***Piptadenia stipulacea*** | |  |  |  |  |  |  |
| JPY584 (D84) | B12 | C3 | *Burkholderia* sp. 4 | JN172099 | JN247656 | JN247655 | NT |
|  |  |  |  |  |  |  |  |
| ***Piptadenia flava*** |  |  |  |  |  |  |  |
| UPRM8060 | R10 | C8 | *R. gallicum* | HE983706 | HE983772 | HE983821 | NT |
| UPRM8061T1 | R10 | C8 | *R. gallicum* | HE983705 | HE983771 | AY166848 | NT |
|  |  |  |  |  |  |  |  |
| ***Anadenanthera peregrina*** | |  |  |  |  |  |  |
| STM7420 | R1 | NT | *Rhizobium* sp*.* 1 | HE983683 | HE983735 | NT | NT |
| STM7426 | R1 | NT | *R. tropici* | HE983686 | HE983738 | NT | NT |
| SMF466_6 | R1 | C12 | *Rhizobium* *leucaenae* | HE983653 | HE983767 | HE983807 | HF536727 |
| IIIA_10R | B13 | C3 | *B. sabiae* | HE983644 | HE983758 | HE983815 | HF536747 |
| STM7419 | B1 | C3 | *B. sabiae* | HE983675 | HE983727 | HE983802 | HF536749 |
| STM7384 | B1 | C3 | *B. sabiae* | HE983682 | HE983734 | HE983804 | HF536748 |
| SMF362_15 | B2 | C5 | *B. nodosa* | HE983648 | HE983762 | HE983809 | NT |
| STM7399 | B8 | C3 | *B. diazotrophica* | HE983685 | HE983737 | HE983785 | HF536745 |
| SMF362_13 | B14 | NT | *B.* c*aribensis* | HE983632 | HE983746 | NT | HF536755 |
| IIIA_4A | B10 | C3 | *B. phenoliruptrix* | HE983643 | HE983757 | HE983808 | HF536740 |
| STM7437 | B7 | C4 | *B. phenoliruptrix* | NT | NT | HE983801 | HF536736 |
| STM7415 | B7 | C4 | *B. phenoliruptrix* | HE983684 | HE983736 | HE983803 | HF536733 |
|  |  |  |  |  |  |  |  |
| ***Anadenanthera colubrina*** | |  |  |  |  |  |  |
| AngicoI_417 | R1 | C12 | *Rhizobium* sp*.* 2 | HE983639 | HE983753 | HE983816 | HF536728 |
| STM7444 | B15 | C3 | *B. diazotrophica* | HE983688 | HE983741 | HE983800 | NT |
| STM7439 | B16 | C3 | *B. diazotrophica* | HE983689 | HE983742 | HE983793 | HF536743 |
| STM7445 | B17 | C3 | *B. diazotrophica* | HE983690 | HE983743 | HE983792 | HF536744 |
| STM7443 | B18 | NT | *B. diazotrophica* | HE983691 | HE983744 | NT | NT |
| STM7452 | B19 | C3 | *B. diazotrophica* | HE983692 | HE983745 | HE983791 | HF536739 |
| STM7454 | B7 | C4 | *B. phenoliruptrix* | HE983687 | HE983740 | HE983799 | HF536735 |
|  |  |  |  |  |  |  |  |
| ***Parapiptadenia pterosperma*** | | |  |  |  |  |  |
| STM7365 | B1 | NT | *B. sabiae* | HE983674 | HE983726 | NT | NT |
| STM7373 | B1 | C1 | *B. sabiae* | HE983678 | HE983730 | HE983790 | HF536757 |
| CVRDII_2 | B20 | C3 | *B. phymatum* | HE983635 | HE983749 | HE983819 | HF536742 |
| STM7363 | B9 | C5 | *B. nodosa* | HE983672 | HE983724 | HE983786 | HF536766 |
| STM7358 | B2 | C5 | *B. nodosa* | HE983673 | HE983725 | HE983788 | HF536760 |
| BR9001 | B2 | NT | *B. nodosa* | HE983640 | HE983754 | NT | NT |
| BR9002 | B9 | C5 | *B. nodosa* | HE983641 | HE983755 | HE983811 | NT |
| SMF142_3 | B2 | NT | *B. nodosa* | HE983649 | HE983763 | NT | NT |
| BR9003 |  | C5 | *B. nodosa* | HE983704 | HE983773 | HE983820 | NT |
|  |  |  |  |  |  |  |  |
| ***Parapiptadenia rigida*** | |  |  |  |  |  |  |
| P.rigida_2 | B1 | C1 | *B. sabiae* | HE983650 | HE983764 | HE983818 | HF536752 |
| UYPR3.611 |  | C1 | *B. sabiae* | JF683695 | ND | JF683727 | NT |
| UYPR1.313 |  | C1 | *B. caribensis* | JF683691 | ND | JF683726 | NT |
| UYPR7.63 |  | C13 | *R. mesoamericanum* | JF683705 | ND | JF683732 | NT |
| BR9004 |  | C5 | *B. nodosa* | JN247665 | JN247657 | JN247658 | NT |
|  |  |  |  |  |  |  |  |
| ***Microlobius foetidus*** | |  |  |  |  |  |  |
| STM7379 | R1 | C12 | *Rhizobium tropici* | HE983679 | HE983731 | HE983776 | NT |
| STM7378 | R6 | NA | *Bradyrhizobium* sp*.*4 | HE9836780 | HE983732 | NA | HF536762 |
| STM7375 | R8 | C10 | *Bradyrhizobium* sp*.*2 | HE983681 | HE983733 | HE983783 | NT |
|  |  |  |  |  |  |  |  |
| ***Pseudopiptadenia contorta*** | |  | |  |  |  |  |
| CVRDIII_5 | B3 | C5 | *B. nodosa* | HE983642 | HE983756 | HE983817 | HF536759 |
| CVRDIII_7 | B2 | C5 | *B. nodosa* | HE983633 | HE983747 | HE983813 | NT |
|  |  |  |  |  |  |  |  |
| ***Pseudopiptadenia psilostachya*** | | |  |  |  |  |  |
| SMF613_4 | R1 | NT | *R. tropici* | HE983634 | HE983748 | NT | NT |

| Reference strains% | Host |  |  | 16S | *recA* | *nodC* | *nifH* |
| --- | --- | --- | --- | --- | --- | --- | --- |
| STM815T | Mimosa |  | *B. phymatum* | NC010622 | NC010622 | NC010622 | NC010622 |
| LMG16225T |  |  | *B. fungorum* | NR_025058 | HQ849138 |  |  |
| LMG19076T |  |  | *B.caledonica* | NR_025057 | HQ849134 |  |  |
| LB400T |  |  | *B. xenovorans* | NC_007951 | NC_007951 |  | NC_007951 |
| AC1100T |  |  | *B. phenoliruptrix* | HQ849094 | HQ849150 |  |  |
| LMG18531T |  |  | *B. caribensis* | NR_026462 | AY644639 |  |  |
| STM678T | Cyclopia |  | *B. tuberum* | AJ302311 | AY644642 | AJ306730 | AJ302315 |
| Br3407T | Mimosa |  | *B. sabiae* | AY533862 | EU294397 |  |  |
| JPY268 | Mimosa |  | *Burkholderia* sp. 2 | FN543672. | FN543815 | FN543534 | FN543955 |
| JPY461T | Mimosa |  | *B. diazotrophica* | FN543755 | FN543898 | FN543616 | FN544034 |
| Br3437T | Mimosa |  | *B. nodosa* | NR_043181 | EU294398 |  |  |
| JPY277 | Mimosa |  | *B. nodosa* | FN543677 | FN543820 | FN543539 | FN543960 |
| BR3470 | Mimosa |  | *B. nodosa* | AY773198 | HE983774 | HE983823 |  |
| Br3461 | Mimosa |  | *B. nodosa* | AY773192 | HE983775 | HE983822 | AY533866 |
| STM3621 | Mimosa |  | *B. mimosarum* | FN908408 | FR669124 | FR853108 | FN908428 |
| PAS44T | Mimosa |  | *B. mimosarum* | NR_043167 | EU294396 | EU386155 | AY883420 |
| JPY321 | Mimosa |  | *B. mimosarum* | FN543702 | FN543845 | FN543566 | FN543985 |
| JPY345T | Mimosa |  | *B. symbiotica* | FN543707 | FN543850 |  | FN543990 |
| JPY347 | Mimosa |  | *B. symbiotica* | FN543709 | FN543852 | FN543572 |  |
| ATCC23344T |  |  | *B. mallei* | NC_006348 | NC_006348 | NC_006348 | NC_006348 |
| ATCC25416 |  |  | *B. cepacia* | U96927 | AF143786 |  |  |
| LMG19424T |  |  | *C. taiwanensis* | CU633749 | CU633749 | CU633751 | CU633751 |
| PfO5 |  |  | *Pseudo. fluorescens* | CP000076 | | | |
| H152T |  |  | *R. giardinii* | NR_026059 | HQ394251 |  |  |
| LMG6214T |  |  | *R. galegae* | X67226 | AM182127 |  |  |
| R602spT |  |  | *R. gallicum* | NR_036785 | AY907357 |  |  |
| CFN42T |  |  | *R. etli* | NC_007761 | NC_007761 | NC_004041 | NC_004041 |
| ATCC19358T |  |  | *R. radiobacter* | AJ389904 | FM164311 |  |  |
| CCGE502T |  |  | *R. grahamii* | JF424608 | JF424622 |  |  |
| CCGE501T |  |  | *R. mesoamericanum* | JF424606 | JF424620 |  |  |
| ATCC11325T |  |  | *R. rhizogenes* | AY945955 | AM182126 |  |  |
| CFN299T |  |  | *R. leucaenae* | EU488741 | EU488817 |  |  |
| CIAT899T |  |  | *R. tropici* | NR_026067 | EU488815 |  |  |
| 3841 |  |  | *R. leguminosarum* | NC_008380 | NC_008380 | NC_008378 | NC_008378 |
| LMG21987T |  |  | *Br. betae* | AY372184 | FM253174 |  |  |
| USDA6T |  |  | *Br. japonicum* | U69638 | AM168341 |  |  |
| BTA1T |  |  | *Br. canariense* | AB079633 | AY591553 |  |  |
| CCBAU10071T |  |  | *Br. yuanmingense* | AF193818 | AY591566 |  |  |
| LMG18230T |  |  | *Br. liaoningense* | AF208513 | FM253180 |  |  |
| USDA76T |  |  | *Br. elkanii* | U35000 | AY591568 |  |  |

Abbreviations: B. : Burkholderia, Br : Bradyrhizobium, R. : Rhizobium, C: Cupriavidus, Pseudo.: Pseudomonas, %: T at the end of the strain name indicates a type strain of the species.
